# All-atom protein sequence design using discrete diffusion models

**DOI:** 10.1186/s13321-025-01121-1

**Published:** 2025-12-01

**Authors:** Amelia Villegas-Morcillo, Gijs J. Admiraal, Marcel J. T. Reinders, Jana M. Weber

**Affiliations:** Department of Intelligent Systems, Delft University of Technology, Delft , The Netherlands

**Keywords:** Protein sequence design, All-atom representation, Discrete diffusion models, Generative modeling

## Abstract

Advancing protein design is crucial for breakthroughs in medicine and biotechnology. Traditional approaches for protein sequence representation often rely solely on the 20 canonical amino acids, limiting the representation of non-canonical amino acids and residues that undergo post-translational modifications. This work explores discrete diffusion models for generating novel protein sequences using the all-atom chemical representation SELFIES. By encoding the atomic composition of each amino acid in the protein, this approach expands the design possibilities beyond standard sequence representations. Using a modified ByteNet architecture within the discrete diffusion D3PM framework, we evaluate the impact of this all-atom representation on protein quality, diversity, and novelty, compared to conventional amino acid-based models. To this end, we develop a comprehensive assessment pipeline to determine whether generated SELFIES sequences translate into valid proteins containing both canonical and non-canonical amino acids. Additionally, we examine the influence of two noise schedules within the diffusion process—uniform (random replacement of tokens) and absorbing (progressive masking)—on generation performance. While models trained on the all-atom representation struggle to consistently generate fully valid proteins, the successfully generated proteins show improved novelty and diversity compared to their amino acid-based model counterparts. Furthermore, the all-atom representation achieves structural foldability results comparable to those of amino acid-based models. Lastly, our results highlight the absorbing noise schedule as the most effective for both representations. Data and code are available at https://github.com/Intelligent-molecular-systems/All-Atom-Protein-Sequence-Generation.

**Supplementary Information:**

The online version contains supplementary material available at 10.1186/s13321-025-01121-1.

## Scientific Contribution

This work introduces a discrete diffusion-based framework for protein sequence generation using an all-atom representation, laying the groundwork for extending to non-canonical amino acids and post-translational modifications. Additionally, it provides a comprehensive evaluation pipeline to assess the validity of generated proteins, demonstrating how noise schedules within the diffusion process impact sequence novelty, diversity, and structural foldability.

**Supplementary Information:**

The online version contains supplementary material available at 10.1186/s13321-025-01121-1.

## Introduction

The ability to successfully design proteins enables transformative solutions for medicine, industry, and environmental sciences [1]. By creating novel proteins or optimizing existing ones, we can develop targeted therapeutics, efficient vaccines, and specialized enzymes for industrial and environmental applications [2]. Proteins are macromolecules composed of long chains of amino acids linked by peptide bonds. The specific sequence of these amino acids dictates how a protein folds into its unique three-dimensional structure, which ultimately determines its function [3]. Protein design involves manipulating either the amino acid sequence [4], the three-dimensional structure [5], or both to achieve desired functional properties [6].

Recent computational advancements, particularly AlphaFold2 [7], have addressed a critical limitation in protein design—structural data scarcity—by enabling accurate structure predictions directly from amino acid sequences [8]. This breakthrough facilitates computational protein design based on sequence information alone. Since experimental techniques for structure determination are resource-intensive and often capture only static snapshots of inherently dynamic proteins [9, 10], structure prediction has become an essential tool for advancing protein design.

Generative models—especially diffusion models—have revolutionized protein design [11, 12]. Diffusion models use a noising process to progressively transform data into noise, and then learn to reverse this process to generate new samples [13–15]. Their success in fields such as computer vision [16] and protein design [5] stems from their ability to produce diverse outputs that can be guided toward specific design objectives. For categorical data like amino acid sequences, discrete denoising diffusion probabilistic models (D3PMs) [17, 18] offer an effective alternative, applying noise through transition matrices characterized by uniform transitions or masking strategies.

Protein sequence and small molecule design have been explored using various discrete diffusion models. One method is EvoDiff [19], which employs D3PMs to generate amino acid sequences through a masking-based noising process. Similar approaches, such as discrete flow models (DFMs) [20] and diffusion optimized sampling (NOS) [21], also explore discrete diffusion for canonical amino acid representations. In contrast, the functional-group-based diffusion framework (D3FG) [22] combines discrete and continuous diffusion for small molecule generation conditioned on a protein target, focusing on 25 functional groups—key chemical substructures that drive molecular properties—and single-atom linkers.

Existing protein sequence design methods rely on conventional amino acid-level representations, where proteins are encoded as strings of the 20 canonical amino acids found in nature [4]. This approach reflects biological processes in which ribosomes translate mRNA sequences into polypeptide chains [23]. However, this traditional representation has limitations, such as its inability to account for all possible non-canonical amino acids [24] or residues that undergo post-translational modifications (PTMs) [25], which can diversify protein functions.

To address these limitations, we propose leveraging all-atom chemical representations for protein sequence design. Beyond including non-canonical amino acids, the all-atom sequence representation allows for modeling protein-small molecule interactions within a shared representation space. This capability is particularly valuable for designing protein binding pockets [26] conditioned on interacting small molecules, with applications in antibody engineering, enzyme optimization, and biosensor development [27–29]. Moreover, this approach facilitates the design of optimized ligands targeting specific proteins, advancing applications in protein-conditioned drug discovery [30, 31].

One promising all-atom approach is self-referencing embedded strings (SELFIES) [32], a string-based molecular representation designed for use in generative models. SELFIES can represent molecules of varying complexity, including longer and more intricate structures [33], while ensuring that every generated sequence corresponds to a chemically valid molecule—an essential feature for protein design. All-atom representations have already demonstrated success in generating small molecules [34], polymers [35], and therapeutic peptides [36]. However, for large protein design tasks, only one existing study has employed SELFIES in generative pre-trained transformers (GPTs), training on canonical amino acids and datasets incorporating molecular fragments for non-canonical amino acids [37]. While this method showed promise in generating diverse proteins, it relied on an auto-regressive model that generates sequences sequentially, therefore not benefiting from the efficiency of parallel generation in diffusion models.

This work introduces the first all-atom diffusion model for large protein design, integrating the SELFIES representation within the discrete diffusion D3PM framework. By representing every atom in the protein backbone and side chains, our approach establishes a foundation for extending generative diffusion models beyond canonical amino acids. While here we train only on canonical proteins, the SELFIES framework naturally accommodates non-canonical amino acids and PTMs, making this work a first step toward models that can incorporate such modifications. Additionally, we investigate the impact of different D3PM noising processes, such as uniform and absorbing noise, on protein sequence generation.

A key contribution of this work is the evaluation of the generated all-atom sequences. Although the SELFIES-generated sequences are chemically valid, this alone does not ensure that the resulting molecule is a protein. Therefore, we assess whether the SELFIES strings can be converted into valid proteins, and categorize their amino acids as canonical or non-canonical. This evaluation, combined with analyses of protein sequence novelty, diversity, and structural foldability, offers valuable insights into the functional potential of all-atom-generated proteins. Together, these contributions pave the way for the application and understanding of all-atom representations in discrete diffusion models for protein design.

## Methodology

### Dataset

Our diffusion models are trained on the UniRef50 dataset [38], which groups protein sequences with a $50\%$ sequence identity threshold. UniRef50 was selected for its balance between comprehensive coverage and sequence diversity. This ensures the model is exposed to a wide range of protein sequences while avoiding high similarity. We obtained protein sequences from the UniRef50 dataset on May 7th 2024. We kept sequences containing solely the 20 canonical amino acids, since non-standard amino acids are denoted as unknown (X), and can thus not be converted to an all-atom representation. While this decision may bias generation toward canonical amino acids, we find that the all-atom models can, in fact, expand beyond this set and generate sequences containing rare, non-canonical residues. Then, we limited the sequence lengths from 30 to 100 amino acids to avoid excessive sequence length expansion with the all-atom representation. After filtering, the final dataset contains around 14 million protein sequences. We split this dataset into training and validation sets (90/10 ratio) with a balanced distribution of protein sequence lengths. In Supplementary Material B, we present our dataset statistics, including the distributions of amino acid and SELFIES sequence lengths, as well as the frequency of amino acid tokens.

**Fig. 1:**
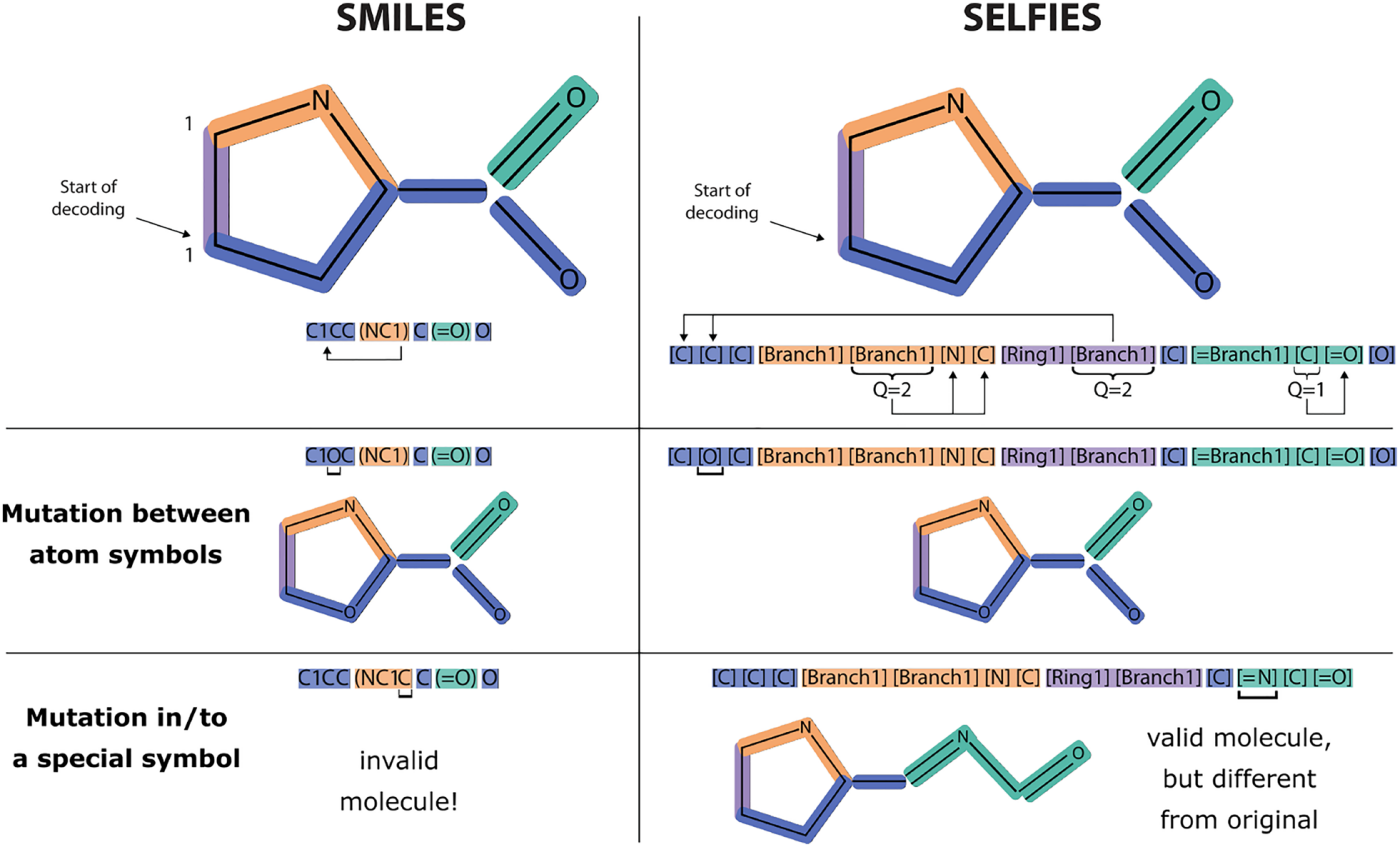
Comparison of SMILES and SELFIES representations for the amino acid Proline. Both examples show how branching and ring formation are handled in their respective representations. In SELFIES, overloaded symbols are annotated with their length or connectivity (Q) to handle these structural features. Decoding starts at the left in both representations and leads to the same canonical SMILES string, which we use in our study. While random mutations in SMILES (especially those involving special symbols) often result in invalid molecules, the SELFIES grammar ensures that all generated sequences decode to valid molecules. (Figure inspired by [32])

### Protein sequence representations

The input to our generative model is either the amino acid sequence tokenized using the 20 canonical amino acids (single-letter codes), or its all-atom representation given by SELFIES [32] (21 tokens). In our case, SELFIES is preferable over other representations, such as SMILES (simplified molecular-input line-entry system) [39], because of its ability to always generate valid molecules (as detailed in Supplementary Material A.1). Fig. 1 illustrates an example of both all-atom representations for the amino acid Proline, showing how random mutations in the symbols affect the resulting molecule. By using SELFIES, we can process the outputs directly, instead of first validating them chemically. Each amino acid sequence is translated to its corresponding SELFIES representation using the Python packages RDKit [40] and SELFIES [41]. The SELFIES grammar compared to the amino acid tokens is shown in Table 1.

**Table 1:** SELFIES tokens used in this research compared to the 20 canonical amino acid tokens

SELFIES tokens (21)	[#Branch1], [#Branch2], [#C], [=Branch1], [=Branch2] [=C], [=N], [=O], [Branch1], [Branch2], [Branch3], [C@@H1] [C@H1], [C], [NH1], [N], [O], [P], [Ring1], [Ring2], [S]
Amino acid tokens (20)	A C D E F G H I K L M N P Q R S T V W Y

### Discrete diffusion model and denoising architecture

We opted to use the discrete denoising diffusion probabilistic model (D3PM) framework due to its competitive performance and the flexibility offered by interchangeable transition probabilities. Furthermore, D3PM enables parallel generation by denoising all sequence positions simultaneously at each step. To model the conditional probability of the reverse process, $p_\theta ({\hat{x}}_0|x_t)$, we chose the ByteNet neural network architecture [42], which demonstrated promising results in protein sequence design for EvoDiff [19], particularly in handling long sequences efficiently and capturing long-range dependencies. More details on the ByteNet architecture can be found in Supplementary Material A.2.

#### Discrete denoising diffusion probabilistic models (D3PMs)

D3PM [18] is a discretized generalized version of DDPM [14] (details in Supplementary Material A.3). In D3PM, the forward process for a scalar random variable with *K* categories ($x_t, x_{t-1} \in 1, \ldots , K$) is defined by a probabilistic transition matrix, represented as $[{\varvec{Q}}_t]_{ij} = q(x_t=j \mid x_{t-1} = i)$. When we denote the row vector ${\varvec{x}}$ as its one-hot version, we can write

1 $$\begin{aligned} q({\varvec{x}}_t \mid {\varvec{x}}_{t-1}) = \text {Cat}({\varvec{x}}_t; {\varvec{p}} = {\varvec{x}}_{t-1}{\varvec{Q}}_t), \end{aligned}$$

where $\text {Cat}({\varvec{x}};{\varvec{p}})$ is a categorical distribution over the one-hot row vector ${\varvec{x}}$ with probabilities given by the row vector ${\varvec{p}}$, and ${\varvec{x}}_{t-1}{\varvec{Q}}_t$ is a row vector-matrix product. From this notation, we derive the two criteria necessary for a noise distribution in a diffusion process:

2 $$\begin{aligned} & q\left( {\varvec{x}}_{t} \mid {\varvec{x}}_0\right) = \text {Cat}({\varvec{x}}_t; {\varvec{p}} = {\varvec{x}}_{0}\bar{{\varvec{Q}}}_t), \text {with } \bar{{\varvec{Q}}}_t={\varvec{Q}}_1{\varvec{Q}}_2 \ldots {\varvec{Q}}_t \end{aligned}$$

3 $$\begin{aligned} & q\left( {\varvec{x}}_{t-1} \mid {\varvec{x}}_t, {\varvec{x}}_0\right) =\frac{q\left( {\varvec{x}}_t \mid {\varvec{x}}_{t-1}, {\varvec{x}}_0\right) q\left( {\varvec{x}}_{t-1} \mid {\varvec{x}}_0\right) }{q\left( {\varvec{x}}_t \mid {\varvec{x}}_0\right) } \nonumber \\ & =\operatorname {Cat}\left( {\varvec{x}}_{t-1}; p=\frac{{\varvec{x}}_t {\varvec{Q}}_t^{\top } \odot {\varvec{x}}_0 \bar{{\varvec{Q}}}_{t-1}}{{\varvec{x}}_0 \bar{{\varvec{Q}}}_t {\varvec{x}}_t^{\top }}\right) , \end{aligned}$$

here, Eq. 2 shows how the noise for any timestep *t* can be efficiently calculated. Equation 3 describes how the tractable forward posterior can be computed using Bayes’ rule. Using this approach, we can set the transition matrices to any noise schedule. In our study, we focus on two noise schedules (see Table 2): uniform noise, which randomly replaces each token with any other token with equal probability, and absorbing (or masking) noise, where tokens progressively transition to a fixed absorbing state (typically denoted as #). Examples of a uniform and absorbing transition matrix for a random variable with three categories at an arbitrary timestep *t* are shown below. The uniform matrix (Eq. 4) consists of the three core categories with equal transition probabilities to other categories. The absorbing matrix (Eq. 5) includes a fourth, masked category that acts as an absorbing state—once entered, all transitions lead exclusively to this category. For both uniform and absorbing noise schedules, we can set the noising parameter to $\beta _t = (T-t+1)^{-1}$ as given by the original D3PM [18]. As *t* increases, $\beta _t$ increases, and the matrix converges to the specified noise distribution.

4 $$\begin{aligned} & \underset{uniform}{{\varvec{Q}}_t} = \begin{bmatrix} 1-\frac{2\beta _t}{3} & \frac{\beta _t}{3} & \frac{\beta _t}{3} \\ \frac{\beta _t}{3} & 1-\frac{2\beta _t}{3} & \frac{\beta _t}{3} \\ \frac{\beta _t}{3} & \frac{\beta _t}{3} & 1-\frac{2\beta _t}{3} \\ \end{bmatrix} \end{aligned}$$

5 $$\begin{aligned} & \underset{absorbing}{{\varvec{Q}}_t} = \begin{bmatrix} 1-\beta _t & 0 & 0 & \beta _t \\ 0 & 1-\beta _t & 0 & \beta _t \\ 0 & 0 & 1-\beta _t & \beta _t \\ 0 & 0 & 0 & 1 \end{bmatrix} \end{aligned}$$

Lastly, an updated loss function is integrated into the diffusion model. Austin et al. [18] use an alternative hybrid loss function, which leads to improved quality of samples:

6 $$\begin{aligned} L_{\text {hybrid}}&= L_{\text {vb}} + \lambda L_{\text {simple}} \nonumber \\&= L_{\text {vb}} + \lambda {\mathbb {E}}_{q\left( {\varvec{x}}_0\right) } {\mathbb {E}}_{q\left( {\varvec{x}}_t \mid {\varvec{x}}_0\right) }\left[ -\log {\widetilde{p}}_\theta \left( {\varvec{x}}_0 \mid {\varvec{x}}_t\right) \right] , \end{aligned}$$

where they introduce an extra denoising objective for the ${\varvec{x}}_0$-parametrization of the reverse process, that encourages good predictions of the data ${\varvec{x}}_0$ at each timestep. This additional objective corresponds to the cross-entropy term $L_0$ (at $t=1$) in DDPM (Supplementary Material A.3), weighted by the $\lambda$ parameter.

#### Training and generation

During the D3PM training phase, the denoising model takes as input a protein sequence and a diffusion timestep. Protein sequences are tokenized according to the selected representation (either SELFIES or amino acid) and mapped to embedding vectors. The diffusion timesteps *t* are encoded into vectors of the same dimension using sinusoidal positional encoding. The sequence embeddings and diffusion timestep encodings are added element-wise and then fed through several ByteNet blocks. The output of the last ByteNet block is embedded back into the protein sequence representation space using a linear layer.

During the inference or sequence generation process, we start with a fully noised sequence given by the noise schedule. We give this sequence and the timestep *T* as input to our model. We then iteratively, predict $p_\theta (\hat{{\varvec{x}}}_0|{\varvec{x}}_t)$ and calculate its posterior $q\left( {\varvec{x}}_{t-1} \mid {\varvec{x}}_t, \hat{{\varvec{x}}}_0\right)$. Using this posterior, we sample the next timestep ${\varvec{x}}_{t-1}$ from a multinomial distribution and feed it into our model together with timestep $t-1$. After progressing through all timesteps, we end up with $\hat{{\varvec{x}}}_0$. Examples of the generation process on both amino acid and SELFIES sequences for the two noise schedules can be found in Table 2 and Supplementary Material C.

**Table 2:**
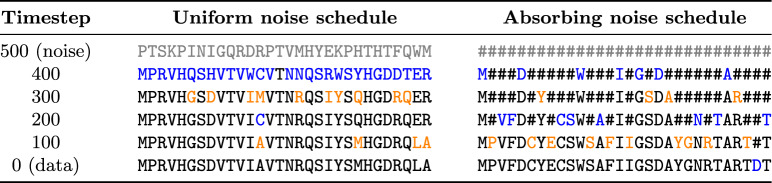
Sequence generation progression for the amino acid representation using both uniform and absorbing noise schedules

Tokens updated at each timestep relative to the previous one are highlighted in alternating blue and orange colors. The absorbing noise process only allows changes from the absorbing state to an amino acid token

### Proposed all-atom-level evaluation and filtering

**Fig. 2:**
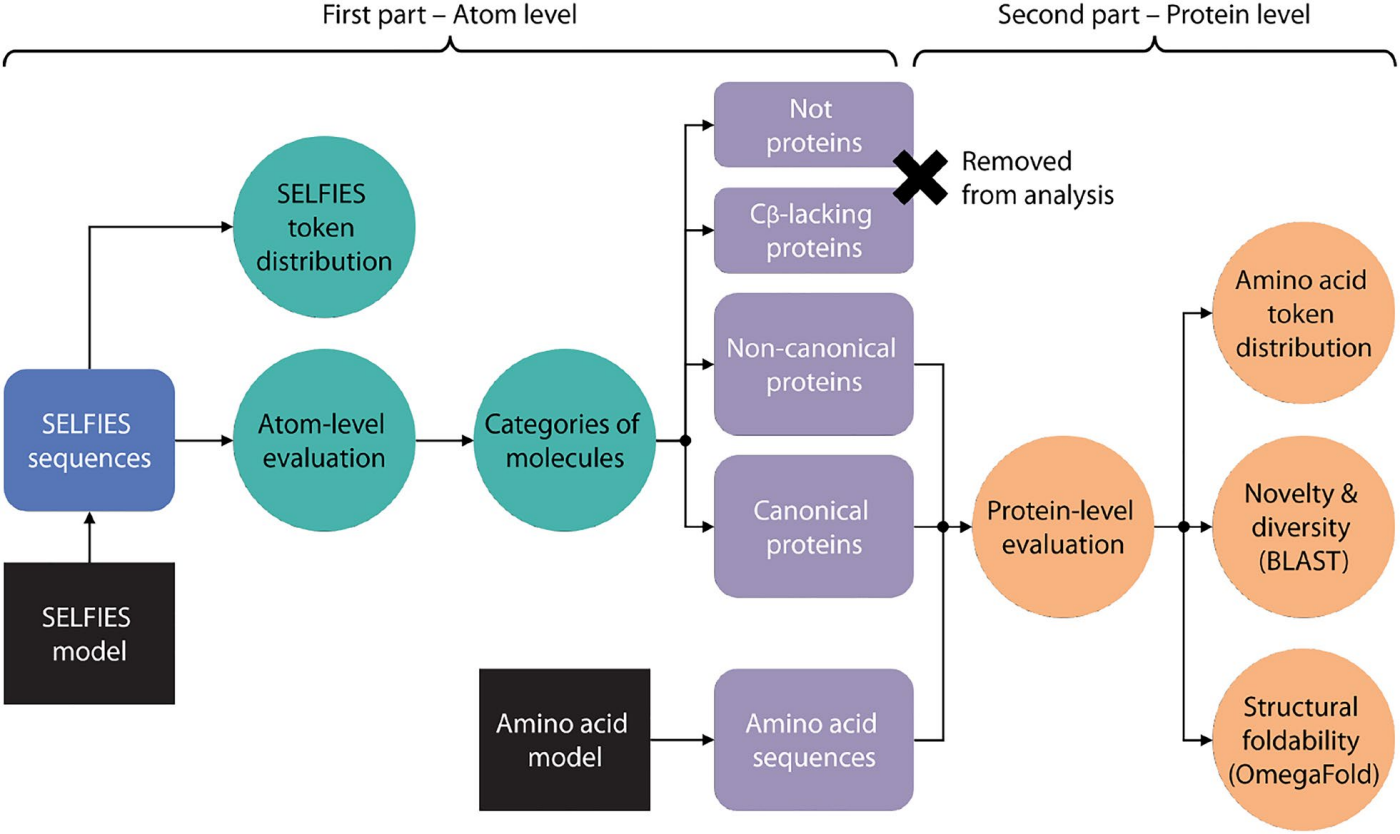
Evaluation workflow for the SELFIES and amino acid generated sequences. All-atom SELFIES sequences are analyzed through various stages, including atom-level evaluation and molecule categorization (in green color), as well as protein-level evaluation of filtered non-canonical and canonical protein sequences (in orange color)

The models trained using the all-atom SELFIES representation focus exclusively on canonical proteins (from UniRef50). However, our experiments revealed that the outputs are not always valid proteins and often include residues beyond the 20 canonical amino acids. While SELFIES ensures chemical validity, this alone does not guarantee that the generated molecules are structurally consistent with real proteins. To assess how well the all-atom model generates protein-like molecules, we developed a set of metrics that evaluate the presence of peptide bonds and a continuous backbone, as well as the constitutional and stereochemistry correctness of the generated amino acids, which we use to construct the amino acid sequence from the SELFIES output. This is the first part of the full evaluation workflow for the all-atom-generated sequences, which is shown in Fig. 2.

*Categorization of generated molecules.* We categorize the generated molecules into the following four classes:

- *Not a protein*: A molecule in which a continuous backbone cannot be constructed or no peptide bonds are present. Such molecules fail to meet the fundamental structural criteria of a protein.
- $C_{\beta }$-*lacking protein:* A protein where at least one amino acid side chain (excluding Glycine) does not begin with a carbon (i.e. lacks a $\beta$-carbon atom). This deviates from both standard amino acid structures and most non-canonical amino acids.
- *Non-canonical protein*: A protein containing non-canonical amino acids or amino acids that are only constitutionally correct but not stereochemically correct. Additionally, molecules failing the SMILES check—indicating extra atoms at the beginning or end of the backbone—are classified as non-canonical.
- *Canonical protein*: A protein composed exclusively of canonical amino acids with correct stereochemistry that passes the SMILES check. This indicates that the generated molecule exhibits all the traits of a canonical protein constructed solely from natural amino acids.

We outline each step of our evaluation pipeline and the corresponding validation criteria below.

*Step 1: Identify peptide bonds and a continuous backbone.* The first step in our analysis involves converting each generated SELFIES sequence into a SMILES string. Since SELFIES guarantees chemical validity, the resulting SMILES also corresponds to a valid molecule. However, the original SELFIES may contain extra tokens that are ignored during decoding. To quantify these unused tokens, we perform a round-trip conversion: each SELFIES sequence is converted to SMILES and then back to SELFIES. Because SMILES only retains atoms and bonds that contribute to a valid structure, this reciprocal check reveals how many tokens in the original SELFIES sequence were effectively discarded.

The SMILES sequence is then transformed into an RDKit [40] molecule graph object for detailed examination. All chemical substructures, including peptide bonds (motif C(=O)-N-C) and side chains, are identified on this explicit molecular graph rather than inferred from raw SELFIES tokens. Our evaluation method relies on performing substructure searches within the molecule and conducting graph traversals of the side chains. Since certain amino acids are substructures of others, directly searching for complete amino acids is ineffective. We begin by *identifying peptide bonds* through substructure searches. Identifying peptide bonds allows us to locate a *continuous backbone* within the molecule. Supplementary Fig. S3 shows a generic amino acid, a peptide bond in a continuous backbone, and two examples of graph traversal on the side chain.

*Step 2: Analyze side-chains, presence of beta-carbon atom, constitutionally and stereochemistry correctness.* Given a continuous backbone and its peptide bonds, we analyze the side chains attached to each $\alpha$-carbon atom. From the $\alpha$-carbon, we find the beginning of the side chain. If no atoms are found, we classify that peptide bond as Glycine, the only amino acid without a side chain. If a side chain is present, we observe the first atom; if it is a carbon atom, we have identified an $\alpha$-$\beta$ carbon bond. Using the RDKit molecule object, we then perform a breadth-first search (BFS) graph traversal of the side chain, exploring new bonds not already part of the backbone or the side chain. If we find a side chain that does not start with a carbon atom, we mark that residue as $\beta$-*carbon lacking* and do not analyze the side chain further. Otherwise, if the atomic structure of the side chain matches one of the 20 natural amino acids, we mark the residue as *constitutionally correct*.

Next, we check for the *stereochemistry correctness* of the generated amino acids. All amino acids, except Glycine, have a chiral center and thus exist as two stereoisomers [43]. These mirror-image forms share similar physical properties in achiral environments but can differ substantially in chemical and biological contexts. Here, we check for amino acids in the *L*-form, as these are the ones found in living organisms.

*Step 3: Build canonical and non-canonical proteins.* If an amino acid meets both constitutional and stereochemistry correctness criteria, we classify it as canonical; otherwise, it is considered non-canonical. We then construct an amino acid sequence for the continuous backbone. Since each side chain was matched to one of the 20 natural amino acids in Step 2, we record canonical residues using their standard one-letter codes and mark non-canonical ones with X (unknown). Finally, we translate the amino acid sequence back into a SMILES representation. By comparing this string to the original SMILES (from the generated SELFIES), we can evaluate whether the model introduced extra atoms at the beginning or end of the backbone (*SMILES check*).

The outcome of this process is the classification of generated molecules into the four categories defined above (and illustrated in Fig. 2). In summary, Step 1 identifies molecules that are not proteins, Step 2 identifies proteins lacking $C_{\beta }$ atoms in their side chains, and Steps 2 and 3 together help classify valid proteins into non-canonical or canonical. By applying this evaluation pipeline, we assess how effectively the all-atom model generates valid proteins and identify where errors occur.

### Protein-level evaluation

We then assess the quality of the generated amino acid sequences using sequence similarity metrics to evaluate novelty and diversity, and 3D structure prediction confidence to estimate structural foldability (second part in Fig. 2).

*Novelty and diversity.* To assess the uniqueness and variability of the generated sequences, we use two metrics: *novelty*, which measures the generation of sequences distinct from the training data, and *diversity*, which quantifies the variation within the generated set.

We use BLAST (basic local alignment search tool) [44] to identify regions of similarity between protein sequences. In BLAST, unknown amino acids (X) are treated as ambiguous residues that can align with any amino acid but do not contribute to the overall alignment score. For each protein search, we record the number of matches, the number of unique matches, and key alignment metrics including the e-value, score, query coverage, and percentage identity. A match is considered significant if its e-value falls below a predefined threshold. For each generated protein set, we also compute the proportion of sequences with at least one significant match. Based on this, we define *novelty* as the proportion of generated sequences with no significant matches to the training dataset, and *diversity* as the proportion of sequences with no significant matches to any other sequence within the generated set. Our initial experiments using a strict e-value threshold of $10^{-5}$ found no significant matches (Supplementary Fig. S4). Therefore, we opted for a threshold of 0.05 to balance between sensitivity and specificity in detecting significant alignments.

*Structure foldability.* We use the structure predictor OmegaFold [45] to evaluate the *structure foldability* of the generated protein sequences. OmegaFold provides a confidence score called pLDDT (predicted local distance difference test) for each residue in the protein, ranging from 0 to 100. In our evaluation, we use pLDDT confidence scores averaged across the whole sequence to assess the structural soundness of the generated proteins. Since OmegaFold does not predict atomic coordinates for unknown amino acids (X), these residues are excluded from the pLDDT calculation. However, their sequence positions are considered, making them appear as gaps in the predicted structure. We consider sequences with average pLDDT scores above 70 reliable and below 50 to be unreliably predicted, following established conventions in protein structure prediction [7]. Scores between 50 and 70 indicate moderate confidence, suggesting the predicted structures may have some correct regions but also areas of high uncertainty.

### Experimental setup

*Implementation and training.* We used the 38M-parameter ByteNet model from EvoDiff. We adapted the original PyTorch implementation to incorporate the SELFIES tokenization and the absorbing noise schedule in the diffusion process. We trained four discrete diffusion models: SELFIES and amino acid representations, uniform and absorbing noise processes. Each model was trained on the UniRef50 dataset with the same architecture and hyperparameters (listed in Supplementary Table S7), using a single NVIDIA A40 GPU. Due to the longer sequence lengths in the SELFIES representation, the total number of tokens in the training set is significantly higher than for the amino acid representation. As a result, the SELFIES models are exposed to more tokens per epoch and converge in fewer epochs (8 epochs, 312 hours), while the amino acid models required 30 epochs (208 hours) to achieve comparable convergence. Training and validation curves can be found in Supplementary Figures S11 and S12.

*Evaluation.* We first evaluated the performance of the all-atom SELFIES models on atom-level metrics for protein filtering and categorization. We generated 1000 sequences of random lengths between the minimum and maximum lengths from the training set (225 to 1907 SELFIES tokens). We refer to this set as *SELFIES (unfiltered)*. We also generated 1000 sequences from the amino acid models (lengths between 30 and 100 amino acids). To ensure a fair comparison, we generated SELFIES sequences until we obtained 1000 non-canonical and 1000 canonical valid proteins. These proteins were filtered to lengths between 10 and 120 amino acids, with no more than 4 non-canonical residues (denoted as unknown X). We refer to these three sets with valid proteins as i. *Amino acid*, ii. *SELFIES (non-canonical)*, and iii. *SELFIES (canonical)*. We then conducted protein-level evaluations using novelty, diversity, and structure confidence as metrics. We additionally run experiments to evaluate the performance across different sequence lengths. This analysis reveals how increasing sequence length affects the models’ ability to generate valid and structurally sound proteins.

Note that our amino acid models correspond to EvoDiff-D3PM [19] (uniform and absorbing variants) trained on our restricted UniRef50 dataset, providing sequence-only baselines directly aligned with the SELFIES setting. As another baseline, we also compare against the amino acid sequence generation method ProtGPT2 [46], which was trained on a much larger dataset of approximately 45M protein sequences from UniRef50. Using this model, we zero-shot generated 1000 canonical proteins following the same sequence length distribution as our training set. Since our training set is smaller and restricted to sequences between 30 and 100 amino acids, differences in novelty, diversity, or structural foldability may be due to the scale and composition of the training set as much as the generative method itself.

## Results and discussion

Our study shows the feasibility of generating valid protein sequences using an all-atom SELFIES representation within discrete diffusion models, marking a step forward in protein design methodologies. In this section, we analyze the performance of the models using both atom-level and protein-level metrics and assess the novelty, diversity, and structural foldability of the generated sequences.

### Absorbing noise enhances validity of SELFIES-generated proteins

We first assess the performance of the all-atom SELFIES models using our proposed atom-level evaluation pipeline. Our analysis reveals that the absorbing model consistently outperforms the uniform model in generating more valid protein sequences.

**Fig. 3:**
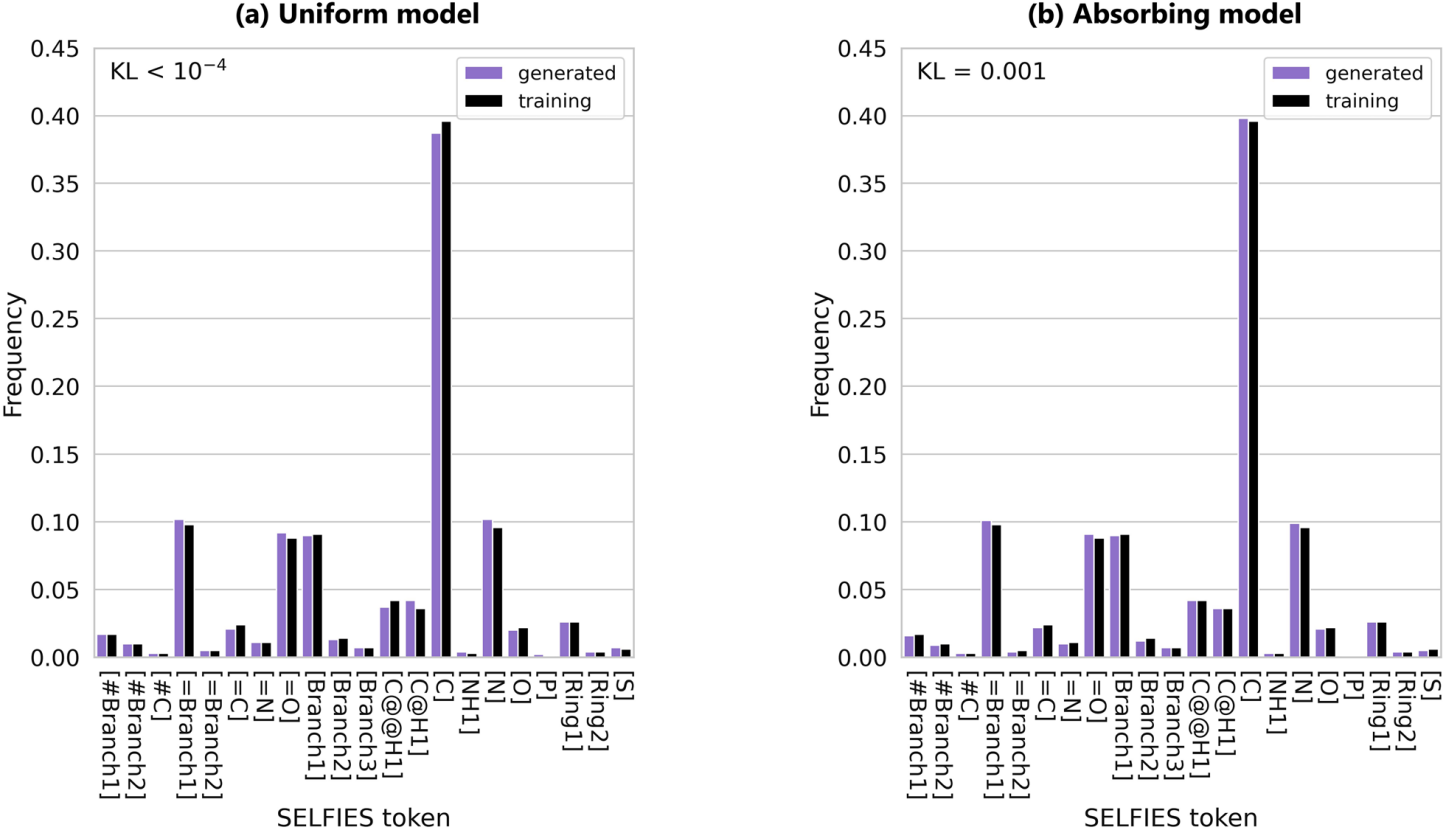
SELFIES token distributions for the 1000 unfiltered all-atom sequences generated by the (**a**) uniform and (**b**) absorbing noise models. Token frequency is calculated as the count of each token divided by the total number of tokens across all generated proteins. We compare to the training set distribution (black bars) and report the Kullback–Leibler divergence, $\textrm{KL}(P_{training} || Q_{generated})$. Both models closely match the training distribution. Notably, the carbon token [C] is the most abundant across all distributions, consistent with organic chemistry

As shown in Fig. 3, both models generate distributions of SELFIES tokens that closely resemble the training set, indicating successful learning of the all-atom vocabulary (see also extended analyses of SELFIES distributions in Supplementary Material D). However, when we analyze how often pairs of SELFIES tokens appear together (2-mer distribution), only 291 out of 441 possible pairs are found in the training set. In contrast, the uniform model generates all 441 pairs, with a KL divergence from the training distribution of 0.034. The absorbing model generates slightly fewer combinations (426), which more closely match the training distribution, with a KL divergence of 0.007. This improved alignment also translates into better performance: the absorbing model outperforms the uniform model by producing sequences with fewer unused tokens, fully continuous backbones, and a higher proportion of constitutionally and stereochemically correct amino acids (Table 3).

**Table 3:** Unused SELFIES tokens and protein categorization for the 1000 unfiltered all-atom SELFIES sequences generated by the uniform and absorbing noise models

	Uniform model	Absorbing model
% Unused SELFIES tokens ($\downarrow$)	55.1%	**18.9%**
% SELFIES sequences with peptide bond ($\uparrow$)	98.0% (980)	**99.5% (995)**
% SELFIES sequences with continuous backbone ($\uparrow$)	5.2% (52)	**23.9% (239)**
% $C_\beta$-lacking proteins (out of cont. backbones) ($\downarrow$)	7.7% (4)	**5.0% (12)**
% Non-canonical proteins ($\uparrow$)	4.4% (44)	**15.0% (150)**
% Canonical proteins ($\uparrow$)	0.4% (4)	**7.7% (77)**

Best results are highlighted in bold

Table 3 shows that the absorbing model generates 239,093 unused SELFIES tokens ($18.9\%$ of the total), compared to 686,273 ($55.1\%$) from the uniform model. The higher unused token ratio observed in the uniform model likely results from its noise process, which allows for token alterations late in the generation process, potentially disrupting sequence coherence. In contrast, the absorbing model does not allow changes to already unmasked tokens, preserving earlier sequence decisions. Moreover, while both models generate sequences with at least one peptide bond, the absorbing model outperforms the uniform model in producing fully continuous backbones (239 compared to 52, out of 1000). This difference highlights the absorbing model’s ability to make fewer errors by using the SELFIES token space more efficiently and maintaining chemical integrity throughout the sequence generation process. Additionally, for the subsets of sequences with continuous backbones, out of the 441 possible 2-mers, we observe 217 (uniform) and 294 (absorbing). These values are closer to the training distribution (291/441), suggesting that constraining generation to empirically observed 2-mers could improve the validity of generated proteins.

In addition, the absorbing model generates more non-canonical and canonical proteins validated by SMILES conversion: 150 and 77, compared to only 44 and 4 from the uniform model. The absorbing model also produces $12/239~(5.0\%)$ $C_\beta$-lacking proteins with continuous backbones, compared to $4/52~(7.7\%)$ in the uniform model. These relatively low occurrences suggest that both models primarily generate sequences with correct amino acid side chains.

**Table 4:** Amino acid-level metrics for all-atom SELFIES sequences with continuous backbones, generated by the uniform and absorbing noise models

	Uniform model	Absorbing model
N. Non-canonical amino acids ($\uparrow$)	93 (8.9%)	**276 (1.9%)**
N. Constitutionally correct amino acids ($\uparrow$)	1180 (90.7%)	**12647 (98.0%)**
N. Stereochemistry correct amino acids ($\uparrow$)	1168 (89.9%)	**12634 (97.9%)**
Non-canonical protein sequence length (avg.±std. $\uparrow$)	24.1 ± 13.3	**60.9** ± **30.7**
Canonical protein sequence length (avg.±std. $\uparrow$)	30.5 ± 7.2	**37.3** ± **15.3**

Average per-sequence ratios are reported in parentheses. The average sequence lengths (in amino acids) are also provided for both non-canonical and canonical protein sets. Best results are highlighted in bold

Out of all the generated SELFIES sequences with continuous backbones (Table 4), the absorbing model produces 12,647 constitutionally correct canonical amino acids, compared to 1180 for the uniform model—a tenfold increase. This suggests that the absorbing model better captures amino acid-level features. While over $90\%$ of amino acids in these sequences are constitutionally correct, the low total count for the uniform model indicates that the generated proteins are very short, as reflected in the relatively low sequence lengths of the non-canonical and canonical proteins (30 amino acids or less on average). Moreover, for both models, the generated canonical amino acids are mostly stereochemically correct, showcasing the models’ ability to capture the stereochemical properties of the dataset.

Next, we examine the results across different SELFIES sequence lengths (number of tokens), as shown in Supplementary Table S3. While both models consistently produce at least one peptide bond per sequence across all length ranges, the number of detected continuous backbones, non-canonical proteins, and canonical proteins decreases as the SELFIES length increases. For the uniform model, the percentage of unused SELFIES tokens increases sharply. This is consistent with the model’s difficulty in producing proteins longer than 40 amino acids on average (Table 4 and Supplementary Table S3), despite being trained on natural proteins up to 100 amino acids. In contrast, the absorbing model shows a more gradual increase in unused SELFIES tokens with sequence length, and it can generate non-canonical proteins even for the longest sequence lengths tested. Notably, the absorbing model produces non-canonical proteins with lengths ranging from 26 to 138 amino acids on average (see Supplementary Table S3), extending beyond the maximum protein sequence length of 100 on which it was trained. This indicates that the absorbing noise process helps generalize to longer valid sequences.

### Absorbing models more accurately reflect amino acid token distributions

We now compare the 1000 sequences generated by the amino acid models (using uniform and absorbing noise schedules) with non-canonical and canonical protein sequences filtered from the SELFIES models. To ensure a fair comparison, we continue generating SELFIES sequences until obtaining 1000 valid proteins of each type. Achieving this required generating 160,000 SELFIES sequences with varying lengths with the uniform model and 20,000 with the absorbing model, re-emphasizing the uniform model’s limited ability to produce fully valid proteins. The resulting amino acid sequence length distributions for each set and noise schedule are shown in Supplementary Fig. S8. The SELFIES models generate proteins outside the range of 30 to 100 amino acids, due to the all-atom representation and the lack of direct control over protein sequence length from the SELFIES strings. Moreover, as previously observed, the SELFIES uniform model tends to generate shorter proteins than the absorbing model, and is unable to produce sequences longer than 70 amino acids.

**Fig. 4:**
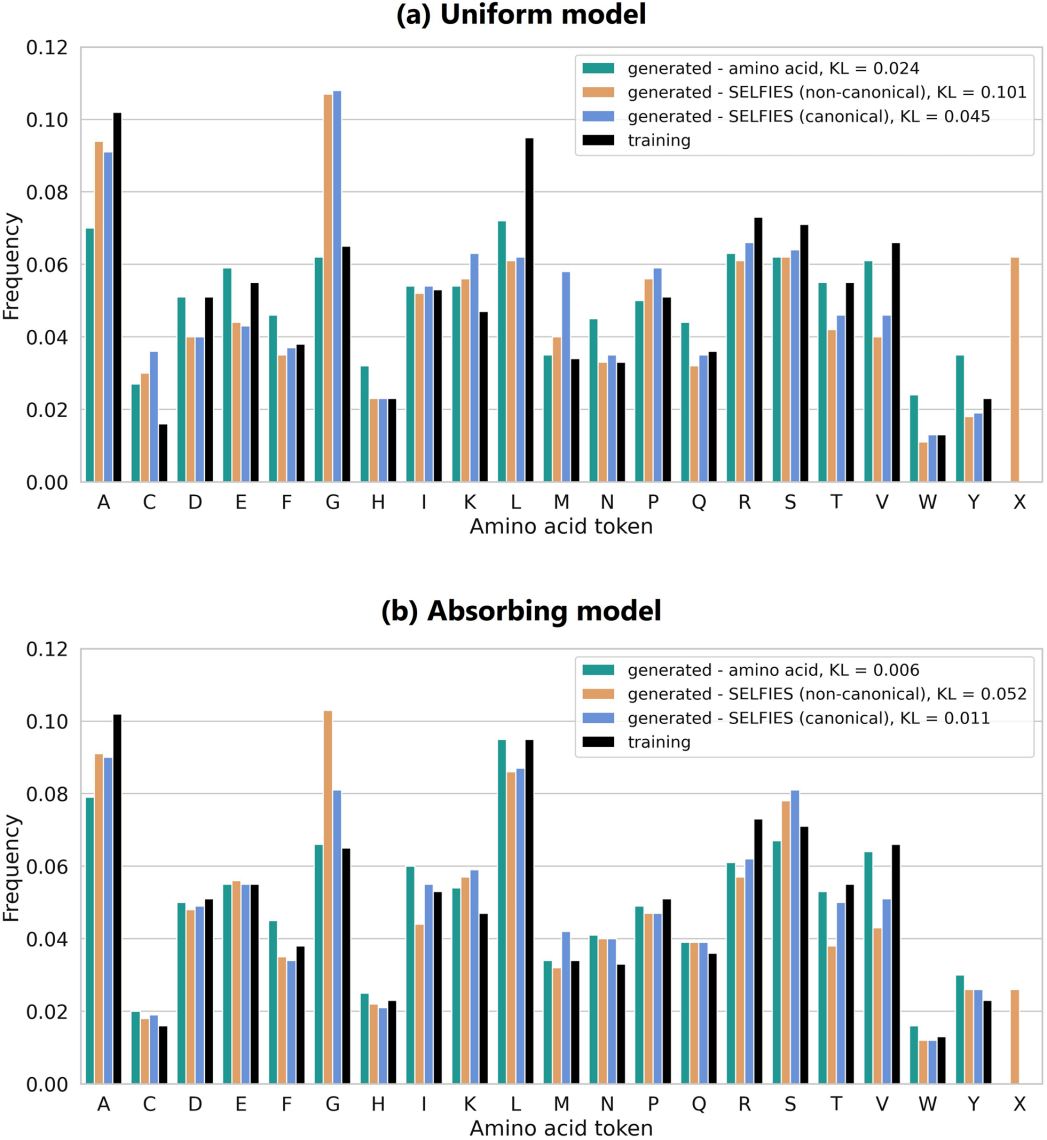
Amino acid token distributions for the 1000 valid proteins in each of the amino acid, SELFIES non-canonical, and SELFIES canonical sets, generated by the (**a**) uniform and (**b**) absorbing noise models. Within each generated set, token frequency is calculated as the count of each token divided by the total number of tokens across all proteins. The X token denotes unknown non-canonical amino acids found exclusively in the generated SELFIES non-canonical proteins. We compare to the training set distribution (black bars) and report the Kullback–Leibler divergence, $\textrm{KL}(P_{training} || Q_{generated})$

The amino acid token distributions for the three sets are illustrated in Fig. 4. The amino acid models, particularly the absorbing one, produce token distributions that closely match the training set, effectively capturing the natural amino acid composition. In contrast, valid proteins generated by the SELFIES models show some deviation from the training distribution; however, these differences remain relatively minor, as reflected by their low Kullback–Leibler (KL) divergence values. In particular, greater deviations—and correspondingly higher KL values—are expected for the non-canonical proteins due to the inclusion of an additional X token, which represents unknown amino acids.

Interestingly, most SELFIES models tend to generate a considerably higher proportion of Glycine (G), the simplest amino acid with no side chain- though this effect is less pronounced in the SELFIES absorbing canonical set. This suggests a bias toward chemically simpler structures, either during generation or as a result of post-processing filters. Conversely, the models underrepresent the amino acids Threonine (T) and Valine (V), despite their side chains not being particularly long. Threonine, in particular, has a branched side chain with a hydroxyl group, introducing both polarity and stereochemical complexity that may make it more challenging to generate correctly.

Across all uniform models, including the amino acid one, we also observe a consistently higher frequency of Cysteine (C) and a lower frequency of Leucine (L), despite Leucine being well represented in the training set. Leucine is chemically similar to Valine, with both having branched nonpolar side chains (Leucine being slightly longer by one methylene group). The lower frequency of both Valine and Leucine may reflect challenges in modeling branched, hydrophobic side chains. In contrast, the elevated occurrence of Cysteine may be due to its simple side chain and distinctive sulfur atom, making it easier for the models to identify or favor during generation.

Although our training data contains only canonical amino acids, we examined whether known non-canonical residues appear in the generated sequences. Comparing against a set of 13 non-canonical amino acids and PTMs representable in our SELFIES vocabulary (PYL, SEP, TPO, PTR, ALY, MLZ, MLY, KCX, CIR, HYP, CME, YCM, and CGU), we found that both the uniform and absorbing models occasionally generate residues corresponding to MLZ (methylated Lysine) and CIR (citrullinated Arginine). While preliminary, these findings suggest that the SELFIES framework can capture chemical variations beyond the canonical set.

### SELFIES models generate highly novel and diverse sequences

Next, we assess the novelty and diversity of sequences generated by the SELFIES models compared to the amino acid models. Overall, the BLAST results indicate that the SELFIES models produce a higher proportion of novel and diverse sequences. Table 5 summarizes these results, reporting the percentage of sequences with no significant matches (e-value below 0.05). Notably, across all models and representations, more than $94\%$ generated sequences lack significant matches, demonstrating consistently high novelty and diversity.

**Table 5:** Percentage of (**a**) novel (e-value $<0.05$ to the training set) and (**b**) diverse (e-value $<0.05$ within the generated set) sequences based on BLAST analysis

Protein set	Uniform model	Absorbing model
**(a) Novelty**		
Amino acid	95.7%	94.6%
SELFIES (non-canonical)	**99.9%**	97.8%
SELFIES (canonical)	99.8%	98.5%
**(b) Diversity**		
Amino acid	94.6%	95.1%
SELFIES (non-canonical)	**99.7%**	98.7%
SELFIES (canonical)	97.8%	96.4%

Results are shown for the uniform and absorbing models, comparing the 1000 valid proteins in each of the amino acid, SELFIES non-canonical, and SELFIES canonical sets. Higher is better for both metrics. Best results are highlighted in bold.

The novelty results (Table 5a) show that proteins generated by the SELFIES models—both non-canonical and canonical—consistently exhibit higher novelty than those produced by the amino acid models. Specifically, the SELFIES uniform model achieves the highest scores, with $99.9\%$ of non-canonical and $99.8\%$ of canonical sequences showing no significant matches to the training set, indicating that nearly all generated proteins are novel. The absorbing SELFIES model also performs well, though with slightly lower novelty. In contrast, the amino acid models yield lower novelty, with a maximum of $95.7\%$ novel sequences for the uniform model, suggesting higher similarity to training data. A similar trend is observed for diversity (Table 5b). The SELFIES uniform model also outperforms, producing highly diverse protein sets: $99.7\%$ of non-canonical and $97.8\%$ of canonical proteins show no significant similarity to other sequences within each generated set. This indicates high sequence variability across the generated proteins. The SELFIES absorbing model also performs well, though diversity among canonical proteins ($96.4\%$) is more comparable to that of the amino acid absorbing model ($95.1\%$). For context, 1000 amino acid sequences generated by the ProtGPT2 baseline model achieve $83.6\%$ novelty and $94.1\%$ diversity. Despite being trained on a much larger dataset, ProtGPT2 shows lower novelty than our amino acid and SELFIES models, while its diversity is similar to our amino acid models but lower than the SELFIES models.

Additionally, when evaluating sequence similarity based on BLAST score, query coverage, and percentage identity (Supplementary Table S5), these metric values are consistently lower for diversity than for novelty. This indicates that, while more matches occur within the generated sets, the sequences are less similar to each other than to those in the training set, which also favors diversity.

### SELFIES absorbing model enhances structural foldability compared to uniform noise

We further investigate the structural foldability of the generated proteins by predicting their 3D structures with OmegaFold. Our results indicate that, while the all-atom SELFIES representation enhances novelty and diversity, this may come at the cost of structural confidence, particularly for longer sequences. Achieving a balance between these properties is crucial for protein design, as highly novel sequences risk being non-foldable structures, compromising their functionality.

**Table 6:** Structural foldability measured as OmegaFold average pLDDT confidence scores. For each set, we report (**a**) the average of all pLDDT scores and (**b**) the percentage of proteins with pLDDT greater than 70 (indicating reliable folding)

Protein set	Uniform model	Absorbing model
**(a) Average pLDDT score**		
Amino acid	53.7 ± 12.7	58.5 ± 13.1
SELFIES (non-canonical)	62.3 ± 12.3	57.0 ± 12.7
SELFIES (canonical)	**64.2** ± **11.9**	62.7 ± 12.5
Train (100k)	66.9 ± 15.3	
**(b) % Proteins with pLDDT** $>70$		
Amino acid	11.0%	19.4%
SELFIES (non-canonical)	27.2%	16.2%
SELFIES (canonical)	**32.1%**	27.9%
Train (100k)	43.6%	

Results are shown for the uniform and absorbing models, comparing the 1000 valid proteins in each of the amino acid, SELFIES non-canonical, and SELFIES canonical sets. We also compare with the pLDDT score of 100k samples from our training set. Higher is better for the pLDDT score. Best results are highlighted in bold

Table 6a shows that, on average, the all-atom SELFIES representation contributes to generating proteins with better predicted structures. Specifically, the canonical proteins generated by the SELFIES uniform model achieve the highest mean pLDDT score of 64.2. Although all models have mean pLDDT scores above 50, none reach the threshold of 70, which is considered indicative of reliable protein folding. However, when examining the percentage of proteins with pLDDT scores above 70 (Table 6b), the SELFIES uniform model generates more foldable proteins than the amino acid models: $32.1\%$ of proteins from the SELFIES canonical set are foldable. Additionally, Supplementary Fig. S9 illustrates the pLDDT score distributions for each model and representation. Notably, almost all generated distributions from the SELFIES models have lower standard deviations than the amino acid models and exhibit higher proportions of foldable proteins (pLDDT $>70$). While these results might suggest a limitation of the models in generating reliably foldable proteins, it is important to consider that the training data also plays a significant role. We observe that even a subset of 100k training proteins does not reach the pLDDT threshold of 70 on average (see distributions in Supplementary Fig. S10). Consistent with this, models trained on much larger datasets face similar limitations. For example, 1000 proteins generated by ProtGPT2 reach an average pLDDT of $64.6 \pm 13.9$, with $38.0\%$ of sequences above the foldability threshold. This underscores that the challenge lies not only in model design but also in the available training data and the accuracy of current structure predictors. Since many sequences in UniRef50 lack experimentally resolved structures, structure predictors like OmegaFold may not accurately assess their foldability—an issue that could also affect the evaluation of generated sequences.

**Fig. 5:**
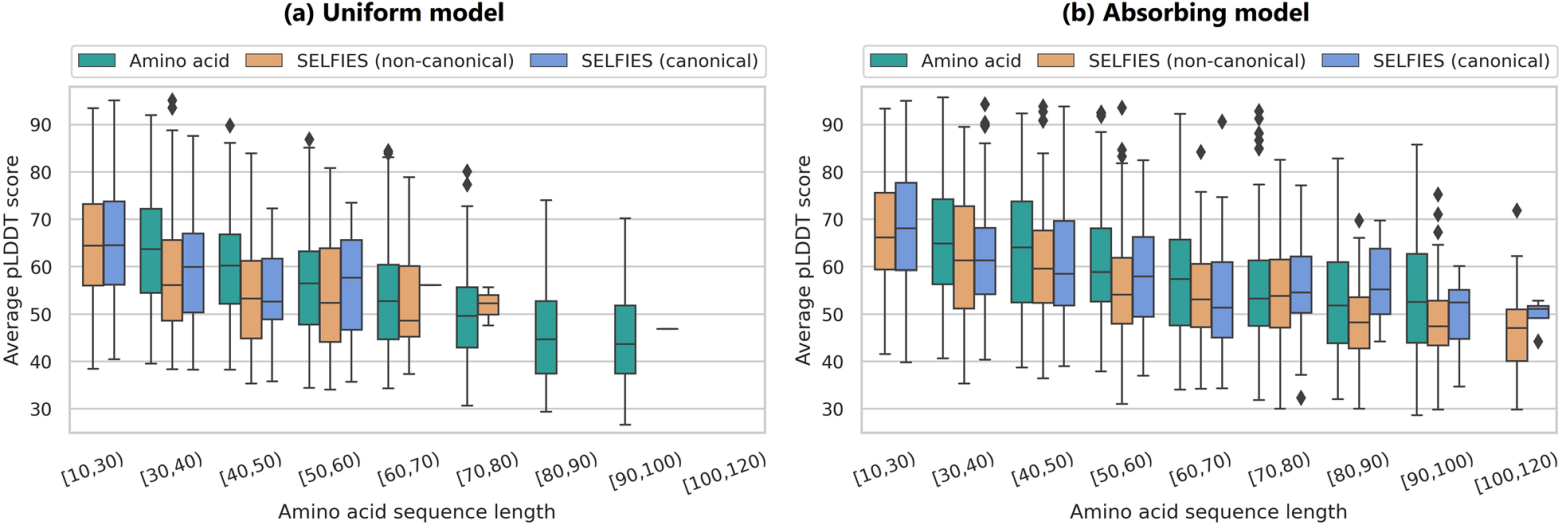
Per-sequence length distribution of average pLDDT confidence scores for the 1000 valid proteins in each of the amino acid, SELFIES non-canonical, and SELFIES canonical sets, generated by the (**a**) uniform and (**b**) absorbing noise models. pLDDT scores are shown in length ranges from 10 to 120 amino acids

When examining pLDDT results for different amino acid sequence lengths (Fig. 5, and Supplementary Table S6 for numerical details), we observe a clear trend: longer protein sequences exhibit lower average pLDDT scores. This is expected, as longer sequences are more complex and prone to cumulative errors during generation, leading to less accurate structures. Furthermore, when we exclude protein sequences shorter than 30 amino acids, the amino acid absorbing model consistently achieves higher average pLDDT scores than the SELFIES models across the different protein length groups. This indicates that the overall average pLDDT result for the SELFIES models is mostly influenced by shorter sequences (less than 30 amino acids) with higher mean pLDDT scores, since short sequences are often trivially foldable. In contrast, the amino acid models were not tasked with generating sequences below 30, resulting in lower means overall. Despite this, the SELFIES absorbing model generates protein sequences across a wide range of amino acid lengths. It achieves the highest mean pLDDT score (68.3) for short canonical proteins (10 to 30 amino acids), approaching the 70 threshold. However, for longer sequences, the amino acid models perform better and yield a higher proportion of foldable proteins (pLDDT $>70$), especially the absorbing model. While the SELFIES absorbing model exhibits slightly weaker performance in terms of structural confidence, it remains a preferred option for applications requiring a broader range of sequence lengths.

## Conclusion

Our work illustrates the significant potential of discrete diffusion models using, for the first time, an all-atom representation for protein sequence generation. This approach offers a more detailed and flexible framework compared to traditional amino acid representations, laying the groundwork for models that can incorporate non-canonical amino acids and post-translational modifications.

While our study primarily focuses on natural protein sequences, the SELFIES representation is expressive enough to accommodate non-canonical amino acids with minor changes in the token vocabulary. This opens an avenue for future research to explore whether such extensions could capture common post-translational modifications or synthetic amino acids used in biocatalysis. This would provide insights into the model’s ability to design synthetic proteins with specialized, enhanced functions.

One challenge of using an all-atom SELFIES representation is that it inflates token sequence length, making it harder for models to capture the long-range dependencies. This contributes to the difficulty of generating longer proteins, particularly for the uniform model. The absorbing model, however, partially alleviates this issue. Combined with the restricted sequence length range of our study (30-100 amino acids), this suggests that future work should explore model architectures and training strategies better suited to longer token sequences.

Another promising direction is to develop interpretability methods for discrete diffusion models in protein generation. For example, analyzing token transition patterns in the uniform model or the dynamics of unmasking in the absorbing model could reveal how chemically valid proteins are constructed during generation and where errors are introduced.

We show that the absorbing SELFIES model, in particular, excels in capturing complex chemical structures and generating novel, diverse sequences, indicating its promise for innovative protein design. However, BLAST analyses do not guarantee biological viability or functional relevance of our generated proteins, and challenges remain in improving their validity and structural reliability.

Addressing these challenges is essential for translating computational designs into functional biological molecules. Future research should focus on refining the all-atom models to reduce the proportion of unusable sequences, addressing biases in amino acid distributions, and enhancing structural stability across varying sequence lengths. By overcoming these limitations, we can advance the design of all-atom proteins through generative models.

## Supplementary Information

Supplementary file 1.

## Data Availability

Data and code for reproducing this work are available at https://github.com/Intelligent-molecular-systems/All-Atom-Protein-Sequence-Generation.

## Abbreviations

BFS: Breadth-first search
BLAST: Basic local alignment search tool
DDPM: Denoising diffusion probabilistic model
D3PM: Discrete denoising diffusion probabilistic model
D3FG: Functional-group-based diffusion model
DFM: Discrete flow model
GPT: Generative pre-trained transformer
GPU: Graphics processing unit
KL: Kullback–Leibler
mRNA: Messenger ribonucleic acid
NOS: Diffusion optimized sampling
pLDDT: Predicted local distance difference test
PTM: Post-translational modification
SELFIES: Self-referencing embedded string
SMILES: Simplified molecular-input line-entry system
