# Supplementary material for: All-atom protein sequence design using discrete diffusion models
Source: J Cheminform. 2025 Dec 1;18:1. doi: 10.1186/s13321-025-01121-1 (PMC12771797; doi:10.1186/s13321-025-01121-1)
Supplement: Supplementary file 1 [file 13321_2025_1121_MOESM1_ESM.pdf]

All-Atom Protein Sequence Design using Discrete Diffusion Models

Supplementary Material

A Background

Here, we present the technical details of various design choices for our research. We first examine the all-atom molecular representations. Next, we discuss the general continuous diffusion model DDPM. Lastly, we present the ByteNet architecture used for the generative diffusion process.

A.1 All-Atom Molecular Representations

Proteins, being chains of bonded amino acids, are intuitively represented by their amino acid sequences. However, they can also be described by their detailed molecular structures. Representing a molecule as a linear string is challenging due to non-linear features like branches and rings. To address this, various techniques [1] have been developed, including SMILES [2], InChI [3], and deep-learning approaches such as DeepSMILES [4] and SELFIES [5].

Simplified molecular-input line-entry system (SMILES) strings [2] have been a prominent method for representing molecular graphs in computational chemistry since 1988. In SMILES, molecules are defined as sequences of atoms represented by letters, with branches denoted by parentheses and ring closures indicated by matching numbers. While SMILES grammar allows for the description of complex structures and properties like stereochemistry and chirality, it is not inherently robust; generative models can produce invalid strings that do not correspond to valid molecular graphs.

To tackle this, self-referencing embedded strings (SELFIES) [5] offer a 100% robust molecular string representation, meaning that any combination of tokens corresponds to a chemically valid molecule. This robustness is achieved because SELFIES are designed to prevent the generation of syntactically and semantically invalid molecules by construction. This property is crucial in generative tasks where producing invalid sequences is undesirable.

In SELFIES, overloading is used to encode chemical structures in a way that eliminates common syntactic errors found in SMILES, such as unbalanced branch parentheses or incorrect ring identifiers. Overloading, in this context, means that certain tokens serve multiple purposes depending on their position and context in the sequence. For example, special tokens like `[Branch1]` or `[Ring1]` initiate branches or rings, and rather than requiring explicit end symbols, the subsequent tokens determine the length and connectivity of these features. This approach simplifies the representation and ensures structural validity throughout the sequence.

Moreover, the SELFIES grammar dynamically tracks the number of available bonds to prevent the generation of semantically incorrect molecules. If a sequence

exhausts the available bonds, the grammar omits further tokens, ensuring the molecule remains chemically valid.

A.2 ByteNet Architecture

ByteNet [6] is a convolutional neural network (CNN) architecture designed for sequence-to-sequence tasks, such as machine translation. The architecture utilizes an encoder-decoder structure, where dilated convolutions are applied in the latent space, allowing the model to capture long-range dependencies within the sequence. Each sequence passes through multiple ByteNet blocks, where dilation functions act as a context window. A context window is the receptive field within which the model can "see" and process surrounding tokens in the sequence. It defines the number of tokens the model considers at a given position, helping it capture dependencies across various ranges without requiring recurrent processing. In each block, the dilation factor denoted as k increases exponentially for each subsequent layer, following the relation: $k = 2^{(n \mod p)}$, where n is the layer index, and $p = \lfloor \log_2 r \rfloor + 1$, with r being the maximum dilation factor at the last block. This exponential growth in dilation allows ByteNet to efficiently cover long contexts in the sequence without increasing the number of layers, which improves the model's efficiency and effectiveness for tasks involving long sequences.

The operations within each ByteNet block are shown in Fig. S1. The layers include normalization (LayerNorm) and activation functions (GeLU), followed by 1×1 convolutions and dilated convolutions with varying dilation factors. These operations ensure

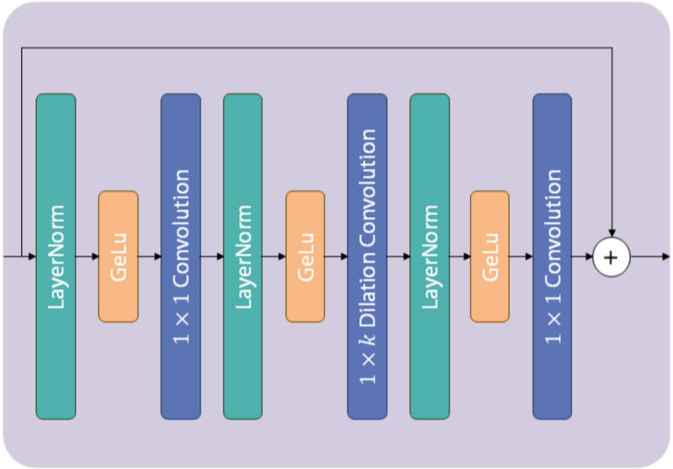

Fig. S1: Schematic of a ByteNet block. Each block includes multiple operations: layer normalization, GeLU activations, 1×1 convolutions, and a key $1\times k$ dilated convolution layer. The dilation factor, k, increases exponentially across layers, allowing the network to capture long-range dependencies. Residual connections are incorporated to aid in model training and gradient flow. (Adapted from [7])

that the network captures both local and long-range features, while the residual connections enable efficient training by allowing information to bypass certain layers, facilitating gradient flow and avoiding vanishing gradients.

ByteNet stands out for its ability to leverage parallel computation across sequences due to its fully convolutional design, making it highly efficient, especially when handling long input sequences. Unlike transformers, which experience quadratic scaling with sequence length and can become computationally intensive. Notably, studies have shown that ByteNet achieves comparable performance to transformers in tasks such as masked protein sequence modeling [7].

A.3 Denoising Diffusion Probabilistic Models (DDPMs)

Three sub-types of diffusion processes exist: denoising diffusion probabilistic models (DDPMs) [8], score-based generative models (SGMs) [9], and stochastic differential equations (SDEs) [10]. Diffusion processes aim to learn data distributions by gradually adding noise to the input and then learning how to reverse this process. While all diffusion models follow this general idea, they differ in how they add and remove noise, and in the architectures they use to reverse the noise. By learning how to effectively denoise corrupted inputs, these models can generate new, realistic data from the learned distributions.

In DDPMs, both the forward and backward processes are defined as Markov chains—a sequence of events where the state of the previous event dictates the probability of the next. A schematic overview of the whole DDPM process is given in Fig. S2.

In the DDPM framework, the forward diffusion process iteratively transforms the original distribution over a specified number of steps, denoted as T. This transformation gradually introduces noise, ultimately converging toward a simpler prior distribution, often a standard Gaussian distribution. The amount of noise added at each step is controlled by a predefined noise schedule, denoted as β_t.

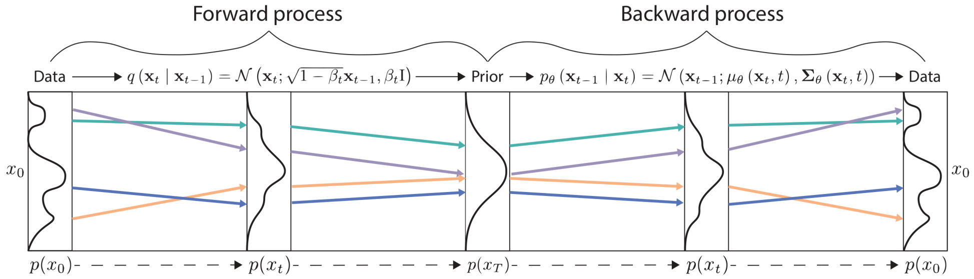

Fig. S2: Schematic of a Denoising Diffusion Probabilistic Model (DDPM) using a continuous Gaussian noising process. This figure illustrates the forward process, which progressively transforms the original complex data into noise, and the backward process, which reverses the noise to generate new data samples. This enables us to generate novel data by learning the underlying distribution of the training data. (Adapted from [10])

Formally, the forward process is defined by the probability $q(\boldsymbol{x}_t \mid \boldsymbol{x}_{t-1})$, where $\boldsymbol{x}_t$ signifies the original input with noise corresponding to timestep t. When a DDPM is used with a continuous Gaussian noise process, its forward process is given as:

$$q(\boldsymbol{x}_t \mid \boldsymbol{x}_{t-1}) = \mathcal{N}(\boldsymbol{x}_t; \sqrt{1-\beta_t}\boldsymbol{x}_{t-1}, \beta_t \boldsymbol{I}). \tag{7}$$

The backward diffusion process uses a neural network architecture with parameters θ that learns to predict the noise added in a forward step. This backward process reconstructs the original input based on the predicted noise at each timestep. The backward process with a Gaussian noise process is given as:

$$p_\theta(\boldsymbol{x}_{t-1} \mid \boldsymbol{x}_t) = \mathcal{N}(\boldsymbol{x}_{t-1}; \mu_\theta(\boldsymbol{x}_t, t), \Sigma_\theta(\boldsymbol{x}_t, t)). \tag{8}$$

To optimize the generative model $p_\theta(\boldsymbol{x}_0)$ and fit it to the data distribution $q(\boldsymbol{x}_0)$, we minimize the variational upper bound on the negative log-likelihood:

$$\begin{aligned} L_{\text{vb}} = \mathbb{E}_{q(\boldsymbol{x}_0)}[&\underbrace{D_{\text{KL}}\left[q\left(\boldsymbol{x}_T \mid \boldsymbol{x}_0\right) \| p\left(\boldsymbol{x}_T\right)\right]}_{L_T} \\ &+ \sum_{t=2}^{T} \underbrace{\mathbb{E}_{q(\boldsymbol{x}_t \mid \boldsymbol{x}_0)}\left[D_{\text{KL}}\left[q\left(\boldsymbol{x}_{t-1} \mid \boldsymbol{x}_t, \boldsymbol{x}_0\right) \| p_\theta\left(\boldsymbol{x}_{t-1} \mid \boldsymbol{x}_t\right)\right]\right]}_{L_{t-1}} \\ &\underbrace{-\mathbb{E}_{q(\boldsymbol{x}_1 \mid \boldsymbol{x}_0)}\left[\log p_\theta\left(\boldsymbol{x}_0 \mid \boldsymbol{x}_1\right)\right]}_{L_0}]. \end{aligned} \tag{9}$$

This equation represents the sum of Kullback–Leibler (KL) divergences between the forward and backward processes at each timestep. The KL divergence measures the statistical distance between a reference and a second probability distribution. The term L_T represents the divergence at the final timestep of the process, while L_0 is the reconstruction loss for the original data sample. The intermediate terms L_{t-1} account for the reconstruction terms between adjacent noising timesteps.

Lastly, selecting an appropriate prior distribution is crucial. The prior distribution must allow for a tractable forward posterior process $q(\boldsymbol{x}_{t-1} \mid \boldsymbol{x}_t, \boldsymbol{x}_0)$ to calculate the KL-divergence loss. Additionally, it must allow efficient computation of $\boldsymbol{x}_t$ from $\boldsymbol{x}_0$ using $q(\boldsymbol{x}_t \mid \boldsymbol{x}_0)$ for any time t. These criteria are met when working with a standard Gaussian noise process.

A.4 Backbone Analysis

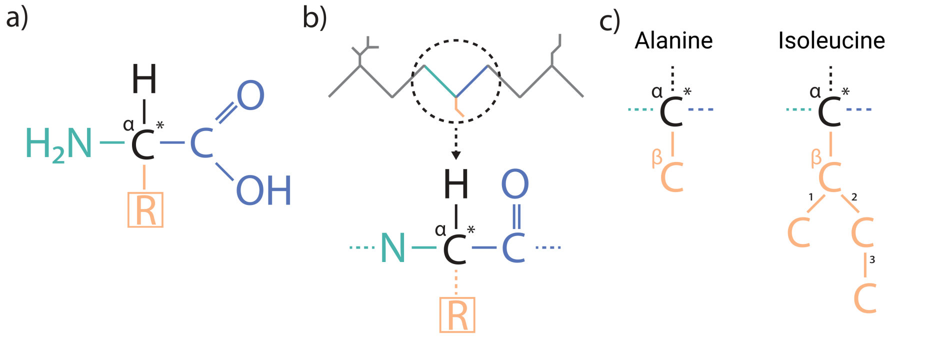

Fig. S3: **(a)** The molecular structure of an amino acid in its non-ionized form, showing the central α-carbon (black), the carboxyl group (blue), the amino group (green), and the variable side chain (orange). The asterisk marks the chiral center. **(b)** A section of a protein backbone highlighting a peptide bond, with the α-carbon indicated as a reference point for side chain analysis. **(c)** Two examples of side-chain structures with α-β carbon bonds: Alanine and Isoleucine. The numbers illustrate the graph traversal order over the side chain bonds, which is used to analyze the molecular structure of the side chain during evaluation. Notably, Alanine can be seen as a substructure of Isoleucine.

B Dataset Analysis

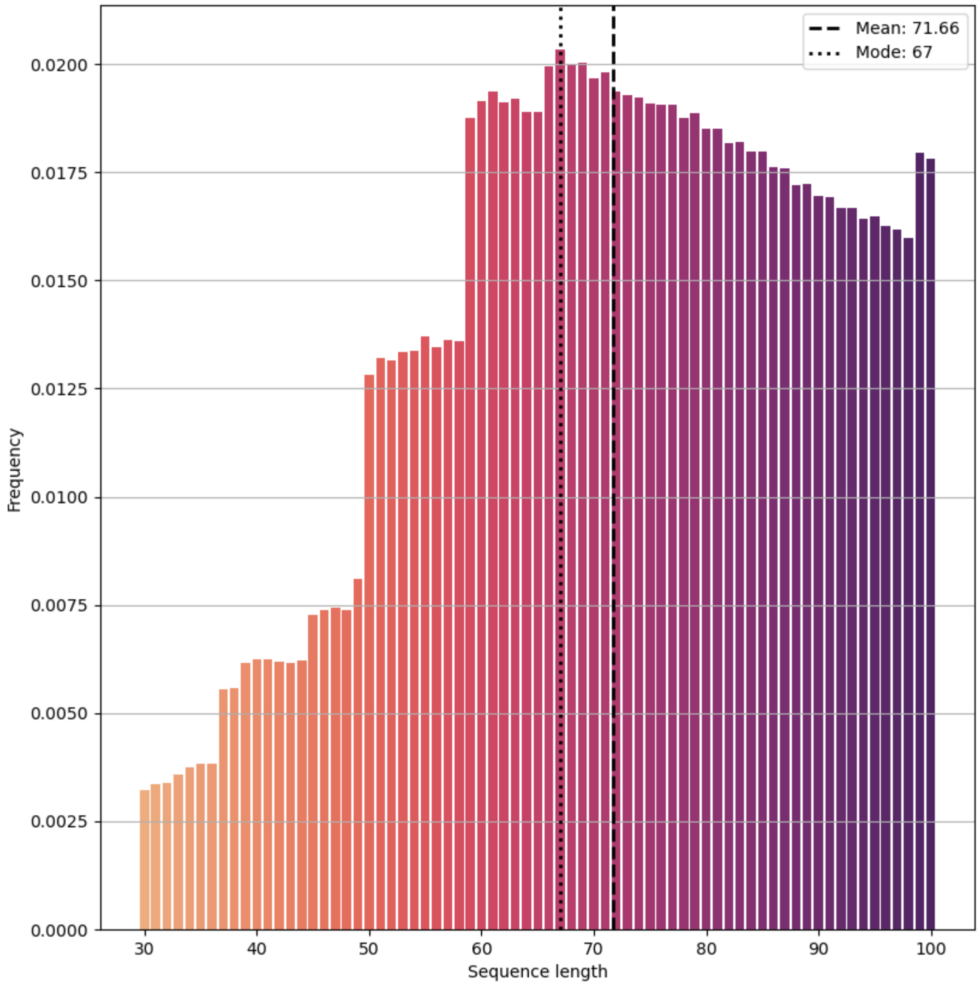

Fig. S4: Distribution of protein sequence lengths in the amino acid representation, ranging from lengths 30 to 100. Both the mean and mode are approximately at length 70, indicating that most proteins in the dataset are around this length. There are relatively few proteins with lengths between 30 and 50. After peaking at length 70, the frequency of sequence lengths declines steadily from 70 to 98. Notably, there is a spike in frequency at lengths 99 and 100.

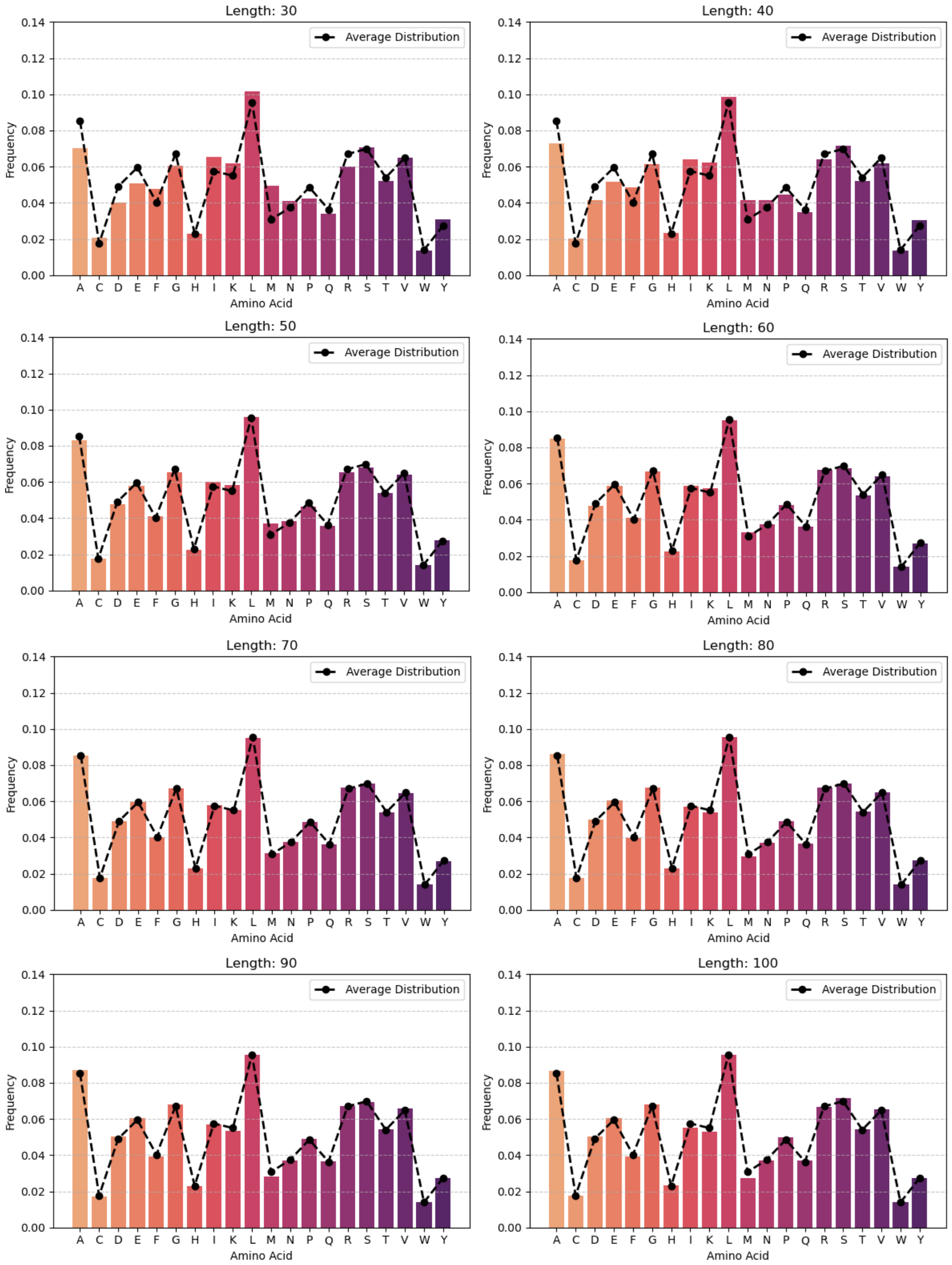

Fig. S5: Amino acid token distributions for protein sequences grouped by length (30–40, 40–50, . . ., 90–100). The dotted line represents the average amino acid distribution for the full dataset. Sequences from 50–100 closely match the average distribution, reflecting their dominance in the dataset. Shorter sequences (30–50) show slight deviations but still reasonably approximate the overall dataset's amino acid composition.

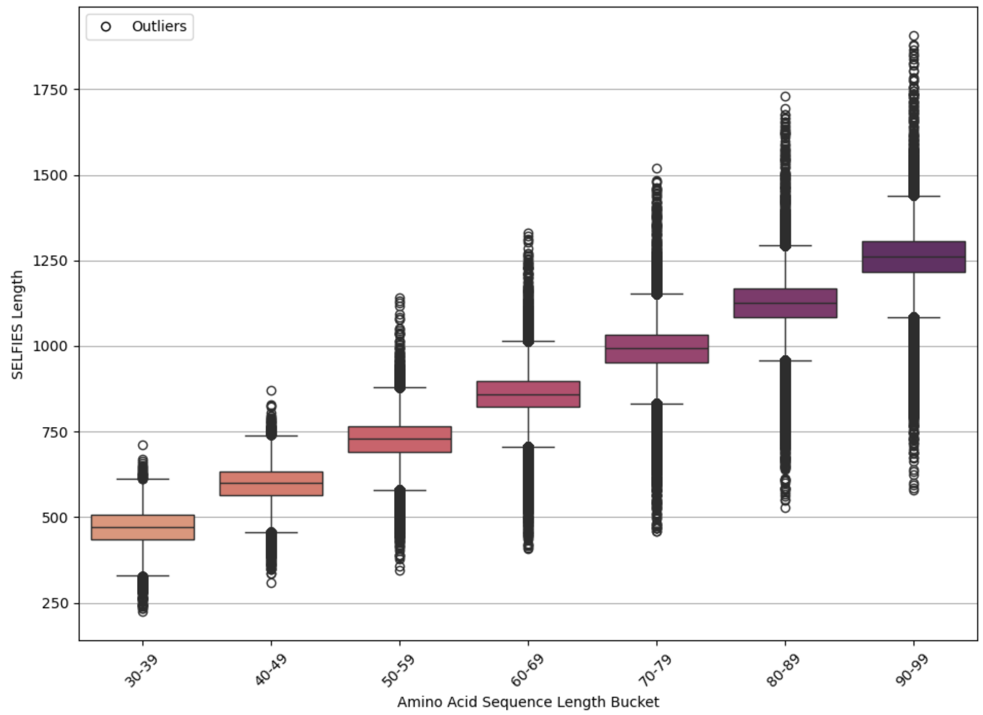

Fig. S6: Distribution of SELFIES lengths by amino acid sequence length buckets. The box plots show that as amino acid sequence length increases, the average SELFIES length scales up approximately linearly. While the spread (interquartile range) increases gradually with longer sequences, indicating a relatively stable distribution, the number of outliers grows more noticeably. This suggests that while longer amino acid sequences generally correspond to longer SELFIES representations, there is an increasing degree of variability at these lengths.

C SELFIES Sequences Denoising Process

Table S1: Sequence generation progression for the all-atom SELFIES representation using the uniform noise schedule.

Step	Uniform noise schedule
500	[#Branch1][Ring2][C@H1][P][P][C@H1][=N][C][P][#C][O][#C][N][N][#C][#Branch1][P][=Branch2][N] ...
450	[S][C][Ring1][=Branch2][#Branch1][C@H1][=Branch2][#Branch1][Ring2][NH1][Ring2][C][C@@H1][=O] ...
400	[C][#Branch2][C@H1][=Branch2][#Branch1][C@H1][Branch3][P][Ring2][Branch1][Ring2][C][S][S][NH1] ...
350	[C][#Branch2][C@H1][Branch1][#Branch1][C@H1][N][Ring1][Ring2][Ring1][Ring2][=O][=O][C@H1] ...
300	[C][NH1][C@H1][Branch1][C][C@H1][N][Ring1][#Branch2][Ring1][Ring2][=O][=O][C@H1][Branch1][=O] ...
250	[C][NH1][C@H1][Branch1][C][C][N][Ring1][Branch3][Ring1][Ring2][=C][=O][Branch2][Branch1][=C][C@H1] ...
200	[C][NH1][C@H1][Branch1][C][C][N][Ring1][Branch3][Ring1][C][=Branch1][=O][Ring2][N][Branch1][C@H1] ...
150	[C][C][C@H1][Branch1][C][C][N][Ring1][Branch3][Ring1][C][=Branch1][=O][=O][N][Branch1][C@H1] ...
100	[C][C][C@H1][Branch1][C][C][N][Branch1][C][Ring1][C][=Branch1][C][=O][N][Branch1][C@H1][C@@H1] ...
50	[C][C][C@H1][Branch1][C][C][C@H1][Branch1][C][Ring1][C][=Branch1][C][=O][N][Branch1][N][=C][#C] ...
0	[C][C][C@H1][Branch1][C][C][C@H1][Branch1][C][N][C][=Branch1][C][=O][N][C@H1][Branch2][=C] ...

Table S2: Sequence generation progression for the all-atom SELFIES representation using the absorbing noise schedule.

Step	Absorbing noise schedule
500	## ...
450	####[C]###[Branch1]###################[C@H1]##################[#Branch2]######################## ...
400	##[C@H1]#[C][C]##[Branch1]####[C]############[C]#[C@H1]#############[=Branch1]#[=O]##[#Branch2] ...
350	##[C@H1]#[C][C]##[Branch1]#[N]##[C]#####[C]######[C]#[C@H1]#############[=Branch1]#[=O]##[#Branch2] ...
300	##[C@H1]#[C][C]##[Branch1]#[N]##[C][=O]####[C]######[C]#[C@H1][Branch1]#####[C]#[C]####[=Branch1] ...
250	##[C@H1][Branch1][C][C]##[Branch1]#[N]##[C][=O]####[C]######[C]#[C@H1][Branch1][#Branch2]###[N][C] ...
200	##[C@H1][Branch1][C][C]##[Branch1]#[N]#[=Branch1][C][=O]####[C]######[C]#[C@H1][Branch1][#Branch2] ...
150	#[C][C@H1][Branch1][C][C]#[Branch2][Branch1]#[N]#[=Branch1][C][=O][C@H1]###[C][C]##[N]##[C]#[C@H1] ...
100	#[C][C@H1][Branch1][C][C]#[Branch2][Branch1]#[N][C][=Branch1][C][=O][C@H1]###[C][C]#[N][N]##[C] ...
50	[C][C][C@H1][Branch1][C][C]#[Branch2][Branch1][=N][N][C][=Branch1][C][=O][C@H1]#[=Branch1][C][C][C] ...
0	[C][C][C@H1][Branch1][C][C][C@H1][Branch2][Branch1][=N][N][C][=Branch1][C][=O][C@H1][Branch1] ...

D Extended SELFIES and All-Atom Results

We performed additional analyses on the token distributions of generated SELFIES sequences after SMILES correction (i.e., from generated SELFIES to SMILES and then back to SELFIES), as well as for subsets with a continuous backbone, non-canonical proteins, and canonical proteins. Fig. S7 shows the extended token distributions for the uniform and absorbing models. As we can see, the SMILES correction occasionally introduces extra tokens in the SELFIES strings not present in the training or original generated sets. The appearance of extra tokens after SMILES correction can be explained by the non-uniqueness of SELFIES: multiple SELFIES strings can represent the same molecule. When round-tripping through SMILES, the representation may change slightly, producing additional tokens. Importantly, this does not affect the chemistry of valid proteins, as the molecular graph remains consistent.

Table S3: Unused SELFIES tokens and protein categorization for the 1000 unfiltered all-atom SELFIES sequences generated by the uniform and absorbing noise models. The results are shown for all SELFIES lengths (number of tokens) and grouped into seven evenly spaced length ranges.

	All lengths	225-465	465-705	705-945	945-1185	1185-1425	1425-1665	1665-1907
**Uniform model**								
% Unused SELFIES tokens (↓)	55.1%	23.2%	35.5%	44.1%	62.5%	68.4%	73.3%	76.9%
N. SELFIES seq. with peptide bond (↑)	980	128	152	139	131	134	150	146
N. SELFIES seq. with continuous backbone (↑)	52	28	12	7	3	1	1	0
N. Non-canonical proteins (↑)	44	24	10	6	3	0	1	0
N. Canonical proteins (↑)	4	3	1	0	0	0	0	0
Non-canonical seq. length (avg.±std. ↑)	24.1±13.3	21.8	21.9	39.0	25.0	–	9.0	–
Canonical seq. length (avg.±std. ↑)	30.5±7.2	27.0	41.0	–	–	–	–	–
**Absorbing model**								
% Unused SELFIES tokens (↓)	18.9%	8.0%	10.9%	13.4%	20.0%	21.8%	25.4%	31.8%
N. SELFIES seq. with peptide bond (↑)	995	142	139	135	157	132	143	147
N. SELFIES seq. with continuous backbone (↑)	239	72	60	47	28	18	7	7
N. Non-canonical proteins (↑)	150	30	31	35	26	17	7	4
N. Canonical proteins (↑)	77	41	27	7	2	0	0	0
Non-canonical seq. length (avg.±std. ↑)	60.9±30.7	26.4	42.0	59.4	81.2	90.2	109.4	138.0
Canonical seq. length (avg.±std. ↑)	37.3±15.3	25.5	45.4	61.9	82.5	–	–	–

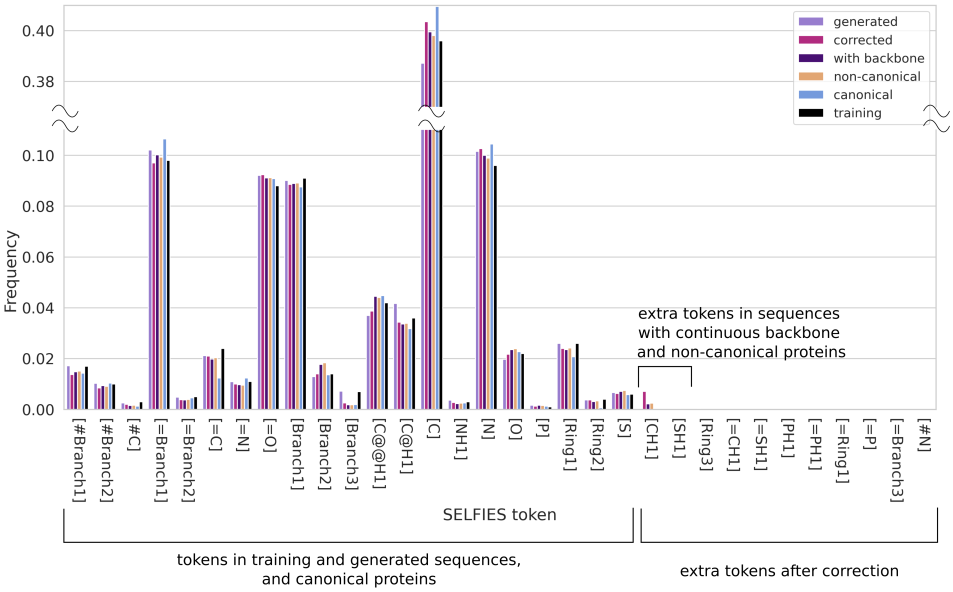

(a) Uniform model

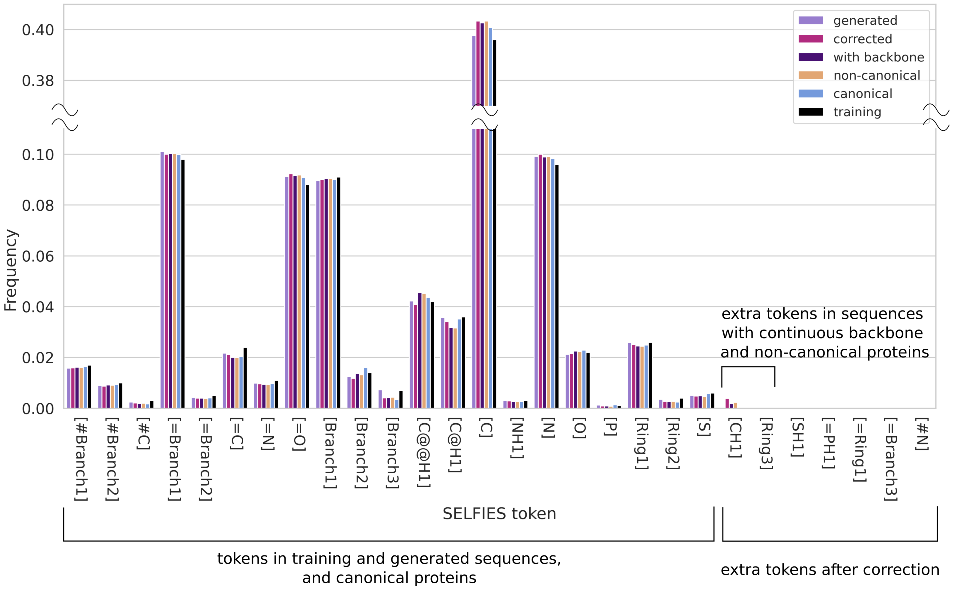

(b) Absorbing model

Fig. S7: SELFIES token distributions for the 1000 unfiltered all-atom sequences generated by the **(a)** uniform and **(b)** absorbing noise models. Token frequency is calculated as the count of each token divided by the total number of tokens across all generated proteins. In addition to the training set distribution (black bars), we compare to the distributions of generated SELFIES sequences after SMILES correction, as well as subsets with a continuous backbone, non-canonical proteins, and canonical proteins.

E Distribution of Amino Acid Sequence Lengths

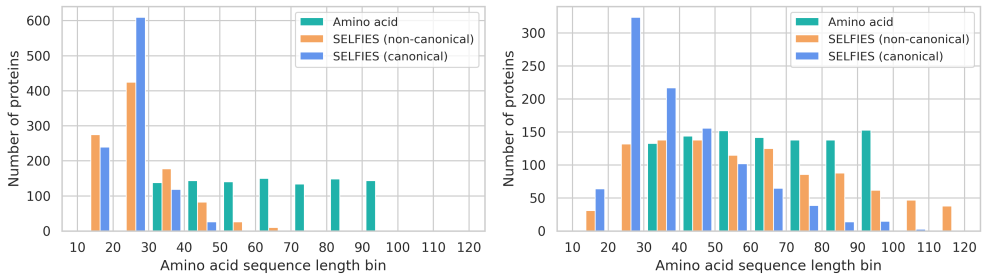

(a) Uniform model

(b) Absorbing model

Fig. S8: Distribution of amino acid sequence lengths for the 1000 valid proteins in each of the amino acid, SELFIES non-canonical, and SELFIES canonical sets, generated by the **(a)** uniform and **(b)** absorbing noise models. Protein counts are shown in evenly spaced bins spanning lengths from 10 to 120 amino acids.

F Extended BLAST Results

Table S4 shows the BLAST results for 1000 amino acid sequences generated using both uniform and absorbing noise processes, evaluated across different e-value thresholds. A match with an e-value $< 10^{-5}$ is considered significant. The table illustrates how, as the e-value threshold becomes less stringent, both the total matches and unique query IDs increase for both noise processes. This analysis informs the selection of an e-value threshold of 0.05 for our final results.

Table S4: BLAST results for the 1000 amino acid sequences generated by the uniform and absorbing noise models, evaluated across different e-value thresholds.

	**Uniform model**				**Absorbing model**			
	**Novelty**		**Diversity**		**Novelty**		**Diversity**	
**e-value threshold**	**Match count**	**Unique IDs**	**Match count**	**Unique IDs**	**Match count**	**Unique IDs**	**Match count**	**Unique IDs**
10^{-5}	0	0	0	0	0	0	0	0
10^{-4}	0	0	0	0	4	2	0	0
10^{-3}	0	0	0	0	31	4	0	0
0.01	6	6	9	9	143	11	10	10
0.05	46	43	54	54	336	54	49	49
0.1	88	86	130	120	535	83	78	74

Table S5: BLAST results for novelty and diversity among the 1000 valid proteins in each of the amino acid, SELFIES non-canonical, and SELFIES canonical sets, generated by the uniform and absorbing noise models. Results are filtered to include matches with an e-value lower than 0.05. The average e-value ranges from 0.015 to 0.03. We also compare with 1000 amino acid sequences generated by the ProtGPT2 baseline model. Lower is better for all the metrics.

	N. Matches (Unique matches)	% Sequences with a match	Score (avg.±std.)	Query coverage (avg.±std.)	Identity (avg.±std.)
**Novelty** (e-value < 0.05)					
ProtGPT2 baseline	13868 (164)	16.4%	105.9 ± 29.9	74.5 ± 17.3	22.2 ± 5.9
**Uniform model**					
Amino acid	46 (43)	4.3%	80.0 ± 2.8	67.9 ± 16.0	16.9 ± 2.6
SELFIES (non-canonical)	1 (1)	0.1%	76.0 ± 0.0	86.8 ± 0.0	15.0 ± 0.0
SELFIES (canonical)	2 (2)	0.2%	76.5 ± 1.5	83.5 ± 2.6	14.5 ± 1.5
**Absorbing model**					
Amino acid	336 (54)	5.4%	81.7 ± 5.2	66.5 ± 15.3	16.5 ± 3.2
SELFIES (non-canonical)	22 (22)	2.2%	81.5 ± 4.6	66.1 ± 19.6	18.9 ± 4.2
SELFIES (canonical)	15 (15)	1.5%	77.6 ± 3.6	80.1 ± 13.8	16.8 ± 3.4
**Diversity** (e-value < 0.05)					
ProtGPT2 baseline	61 (59)	5.9%	53.5 ± 7.2	56.9 ± 18.7	13.8 ± 4.2
**Uniform model**					
Amino acid	54 (54)	5.4%	51.0 ± 3.2	53.1 ± 17.1	11.3 ± 2.4
SELFIES (non-canonical)	3 (3)	0.3%	41.3 ± 1.7	55.4 ± 4.6	8.3 ± 0.9
SELFIES (canonical)	22 (22)	2.2%	40.9 ± 2.2	76.6 ± 12.4	8.5 ± 1.4
**Absorbing model**					
Amino acid	49 (49)	4.9%	50.9 ± 4.4	52.2 ± 19.2	11.1 ± 3.1
SELFIES (non-canonical)	20 (19)	1.9%	50.4 ± 2.9	47.0 ± 21.3	12.1 ± 1.8
SELFIES (canonical)	36 (36)	3.6%	47.0 ± 2.8	60.1 ± 21.8	10.3 ± 2.0

G Extended OmegaFold pLDDT Results

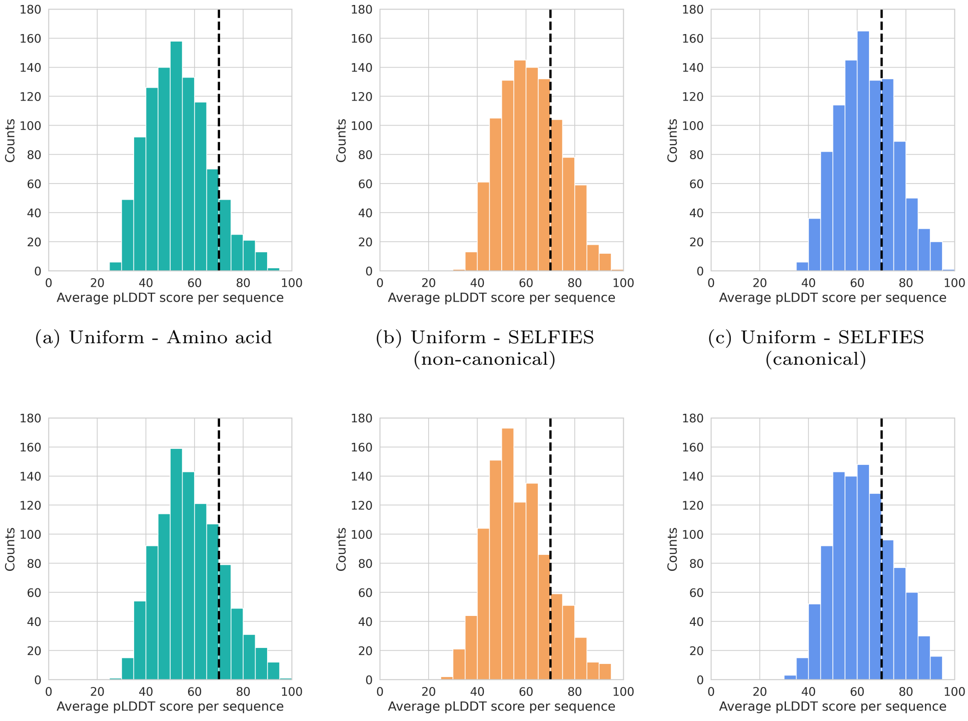

(a) Uniform - Amino acid

(b) Uniform - SELFIES (non-canonical)

(c) Uniform - SELFIES (canonical)

(d) Absorbing - Amino acid

(e) Absorbing - SELFIES (non-canonical)

(f) Absorbing - SELFIES (canonical)

Fig. S9: Distribution of OmegaFold average pLDDT scores for the 1000 valid proteins in each of the amino acid, SELFIES non-canonical, and SELFIES canonical sets, generated by the uniform and absorbing noise models. Dotted lines indicate the pLDDT threshold of 70, above which structure predictions are considered reliable.

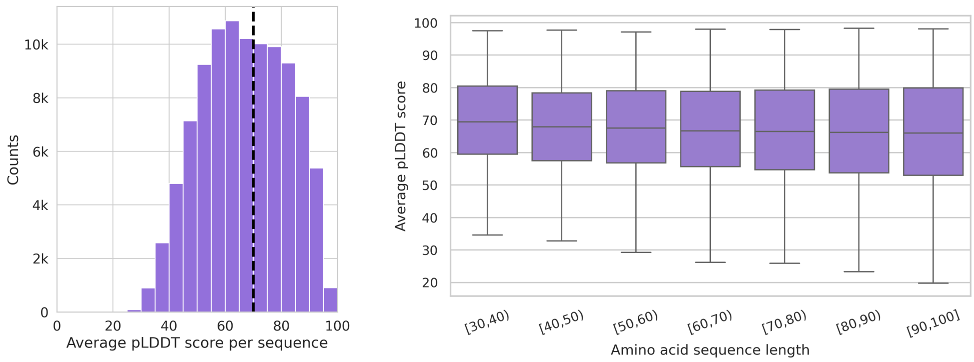

Fig. S10: Distribution of OmegaFold average pLDDT scores for 100k training samples.

Table S6: OmegaFold average pLDDT confidence scores for the 1000 valid protein in each of the amino acid, SELFIES non-canonical, and SELFIES canonical sets, generated by the uniform and absorbing noise models. The results are shown for all lengths and grouped into nine length ranges. The first [10-30) and last [100-120) ranges handle outliers, while the rest are evenly spaced from 30 to 100, following the training set distribution. We also compare with the pLDDT score of 100k samples from the training set, and 1000 amino acid sequences generated by the ProtGPT2 baseline model. Higher is better for the pLDDT score. Best results within our diffusion models are highlighted in bold.

	All (avg.±std.)	[10-30)	[30-40)	[40-50)	[50-60)	[60-70)	[70-80)	[80-90)	[90-100)	[100-120)
**Train (100k)**										
N. Proteins	100k	0	4.2k	6.9k	13.7k	19.5k	19.2k	17.9k	18.5k	0
Avg. pLDDT	66.9±15.3	–	69.5	67.9	67.7	67.0	66.7	66.2	66.0	–
N. pLDDT > 70	43.6k	–	2.1k	3.1k	6.1k	8.4k	8.2k	7.7k	7.9k	–
**ProtGPT2**										
N. Proteins	1000	0	143	143	143	142	143	143	0	
Avg. pLDDT	64.6±13.9	–	68.9	66.4	67.3	64.6	62.9	61.4	60.9	–
N. pLDDT > 70	380	–	72	55	65	55	50	40	43	–
**Uniform model**										
**Amino acid**										
N. Proteins	1000	0	138	144	141	150	134	149	144	0
Avg. pLDDT	53.7±12.7	–	64.0	60.3	56.4	53.6	50.0	46.6	45.0	–
N. pLDDT > 70	110	–	39	27	20	13	5	5	1	–
**SELFIES (non-canonical)**										
N. Proteins	1000	700	178	83	26	10	3	0	0	0
Avg. pLDDT	62.3±12.3	64.7	58.3	54.2	54.0	53.6	51.9	–	–	–
N. pLDDT > 70	272	228	31	9	3	1	0	–	–	–
**SELFIES (canonical)**										
N. Proteins	1000	850	119	26	3	1	0	0	1	0
Avg. pLDDT	**64.2±11.9**	65.3	59.3	54.7	55.6	56.2	–	–	46.9	–
N. pLDDT > 70	**321**	296	21	3	1	–	–	0	–	
**Absorbing model**										
**Amino acid**										
N. Proteins	1000	0	133	144	152	142	138	138	153	0
Avg. pLDDT	58.5±13.1	–	**65.5**	**63.9**	**60.9**	**57.8**	**55.1**	53.0	**53.4**	–
N. pLDDT > 70	194	–	45	47	32	26	16	12	16	–
**SELFIES (non-canonical)**										
N. Proteins	1000	163	138	138	115	125	86	88	62	85
Avg. pLDDT	57.0±12.7	67.3	62.2	60.5	55.7	54.3	53.9	48.9	48.3	46.2
N. pLDDT > 70	162	60	45	29	12	7	6	0	2	1
**SELFIES (canonical)**										
N. Proteins	1000	388	217	156	102	65	39	14	15	4
Avg. pLDDT	62.7±12.5	**68.3**	62.0	61.0	57.8	53.1	**55.1**	**56.3**	50.1	**49.8**
N. pLDDT > 70	279	177	44	39	12	3	4	0	0	0

H Model Hyperparameters

Table S7: Summary of hyperparameter configurations used in our D3PM implementation with the ByteNet architecture, including details on the dataset processing, optimizer settings, and learning rate scheduler. Each hyperparameter is listed alongside its value and source.

**Hyperparameter**	**Value**	**Source**
**ByteNet model**		
Embedding dimension (d_{embed})	8	EvoDiff
Model dimension (d_{model})	1024	EvoDiff
Activation function	GeLU	EvoDiff
Slim	True	EvoDiff
Number of layers (n_{layers})	16	EvoDiff
Kernel size	5	EvoDiff
Max dilation value (r)	128	EvoDiff
Diffusion timesteps	500	EvoDiff
Number of tokens amino acid (n_{tokens})	20	This work
Number of tokens SELFIES (n_{tokens})	21	This work
Loss reweighting uniform (λ)	0	D3PM
Loss reweighting absorbing (λ)	0.1	D3PM
Causal	False	EvoDiff
Dropout	0.1	EvoDiff
Tie weights	False	EvoDiff
Final norm	False	EvoDiff
**Dataset and batch sampling**		
Dataset	UniRef50	This work
Max tokens	40000	EvoDiff
Max batch size	800	EvoDiff
Bucket size	1000	EvoDiff
Max epoch	500	EvoDiff
**Optimizer and scheduler**		
Optimizer	Adam	EvoDiff
Learning rate	10^{-4}	EvoDiff
Weight decay	0	EvoDiff
Scheduler	LambdaLR	EvoDiff
Warm-up steps	10000	EvoDiff

I Training and Validation Loss

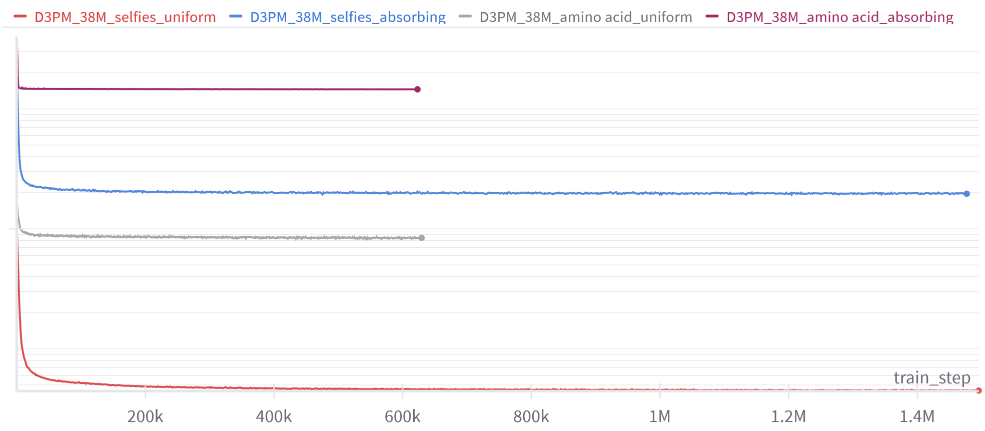

Fig. S11: Training loss per step for the amino acid and SELFIES models under both uniform and absorbing noise schedules. All models have largely converged. (y-axis is in log scale).

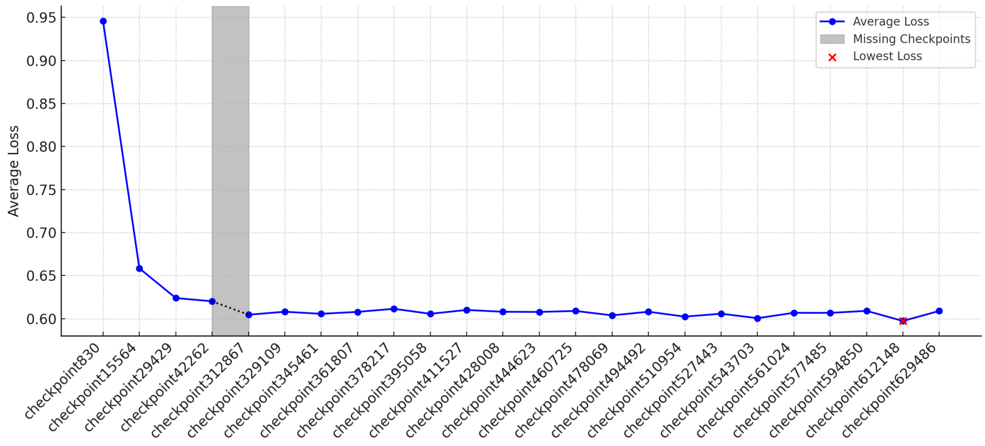

(a) Amino acid model - Uniform noise

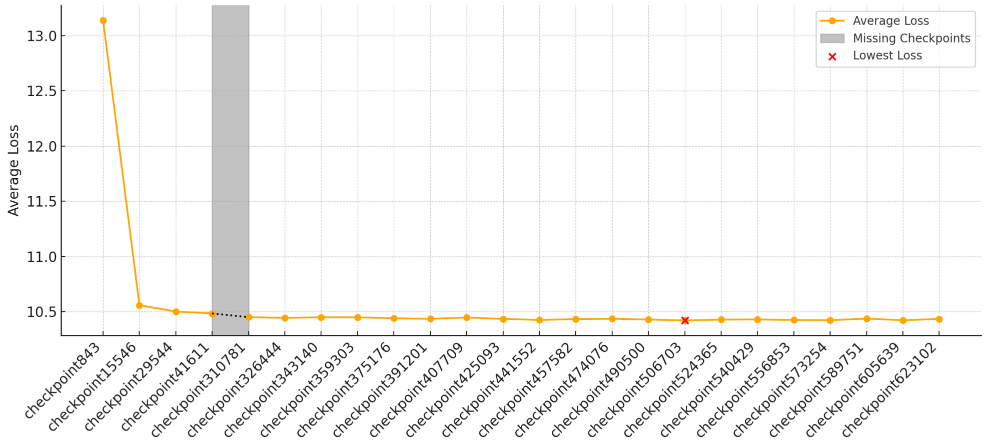

(b) Amino acid model - Absorbing noise

Fig. S12: Average validation loss across different checkpoints for the amino acid and SELFIES models using both uniform and absorbing noise schedule. (Cont.)

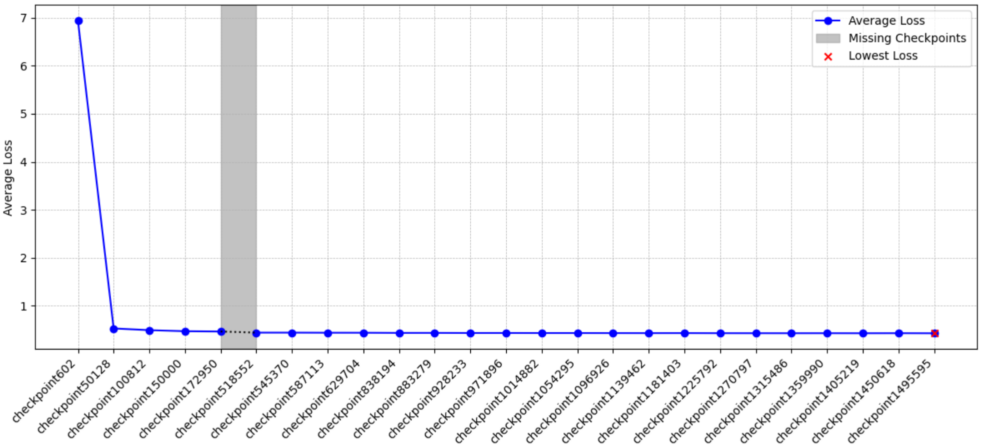

(c) SELFIES model - Uniform noise

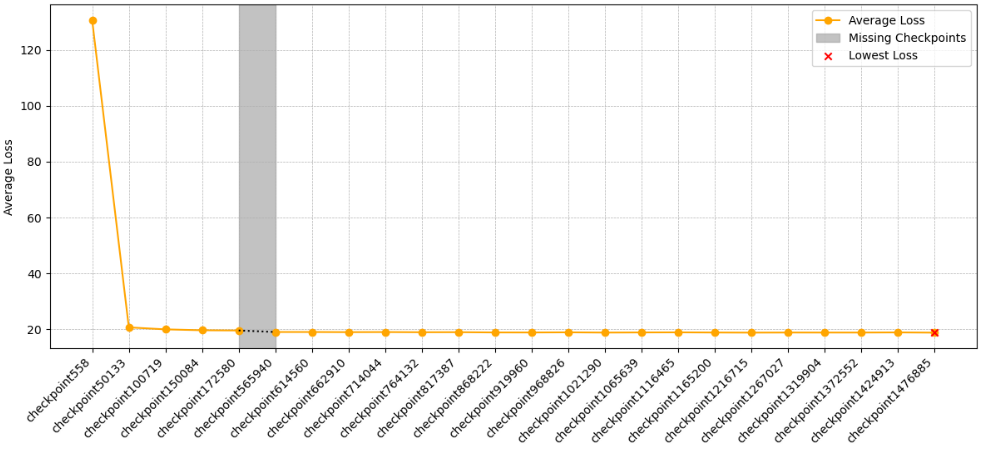

(d) SELFIES model - Absorbing noise

Fig. S12: Average validation loss across different checkpoints for the amino acid and SELFIES models using both uniform and absorbing noise schedule. The lowest average validation loss checkpoint is highlighted, indicating the best-performing model. The trend shows that the models have largely converged, with minimal variation in loss across the later checkpoints.

Supplementary References

[1] Mario Krenn, Qianxiang Ai, Senja Barthel, Nessa Carson, Angelo Frei, Nathan C Frey, Pascal Friederich, Théophile Gaudin, Alberto Alexander Gayle, Kevin Maik Jablonka, et al. SELFIES and the future of molecular string representations. *Patterns*, 3(10), 2022.

[2] David Weininger. SMILES, a chemical language and information system. 1. introduction to methodology and encoding rules. *Journal of Chemical Information and Computer Sciences*, 28(1):31–36, 1988.

[3] Stephen R Heller, Alan McNaught, Igor Pletnev, Stephen Stein, and Dmitrii Tchekhovskoi. InChI, the IUPAC international chemical identifier. *Journal of Cheminformatics*, 7:1–34, 2015.

[4] Noel O'Boyle and Andrew Dalke. DeepSMILES: an adaptation of SMILES for use in machine-learning of chemical structures. *ChemRxiv*, 2018.

[5] Mario Krenn, Florian Häse, AkshatKumar Nigam, Pascal Friederich, and Alan Aspuru-Guzik. Self-referencing embedded strings (SELFIES): A 100% robust molecular string representation. *Machine Learning: Science and Technology*, 1 (4):045024, 2020.

[6] N Kalchbrenner. Neural machine translation in linear time. *arXiv preprint arXiv:1610.10099*, 2016.

[7] Kevin K Yang, Nicolo Fusi, and Alex X Lu. Convolutions are competitive with transformers for protein sequence pretraining. *Cell Systems*, 15(3):286–294, 2024.

[8] Jonathan Ho, Ajay Jain, and Pieter Abbeel. Denoising diffusion probabilistic models. *Advances in Neural Information Processing Systems*, 33:6840–6851, 2020.

[9] Arash Vahdat, Karsten Kreis, and Jan Kautz. Score-based generative modeling in latent space. *Advances in Neural Information Processing Systems*, 34:11287–11302, 2021.

[10] Yang Song, Jascha Sohl-Dickstein, Diederik P Kingma, Abhishek Kumar, Stefano Ermon, and Ben Poole. Score-based generative modeling through stochastic differential equations. *arXiv preprint arXiv:2011.13456*, 2020.
